# On-chip nanophotonic topological rainbow

**DOI:** 10.1038/s41467-022-30276-w

**Published:** 2022-05-11

**Authors:** Cuicui Lu, Yi-Zhi Sun, Chenyang Wang, Hongyu Zhang, Wen Zhao, Xiaoyong Hu, Meng Xiao, Wei Ding, Yong-Chun Liu, C. T. Chan

**Affiliations:** 1 Key Laboratory of Advanced Optoelectronic Quantum Architecture and Measurements of Ministry of Education, Beijing Key Laboratory of Nanophotonics and Ultrafine Optoelectronic Systems, School of Physics, Beijing Institute of Technology, Beijing, 100081 China; 2 Collaborative Innovation Center of Light Manipulations and Applications, Shandong Normal University, Jinan, 250358 China; 3 Institute of Photonics Technology, Jinan University, Guangzhou, 510632 China; 4 State Key Laboratory for Mesoscopic Physics & Department of Physics, Collaborative Innovation Center of Quantum Matter & Frontiers Science Center for Nano-optoelectronics, Beijing Academy of Quantum Information Sciences, Peking University, Beijing, 100081 China; 5 Key Laboratory of Artificial Micro- and Nano-Structures of Ministry of Education, School of Physics and Technology, Wuhan University, Wuhan, 430072 China; 6 State Key Laboratory of Low-Dimensional Quantum Physics, Department of Physics, Frontier Science Center for Quantum Information, Tsinghua University, Beijing, 100084 China; 7 Department of Physics, The Hong Kong University of Science and Technology; Clear Water Bay, Kowloon, Hong Kong, China

**Keywords:** Photonic crystals, Metamaterials, Silicon photonics, Nanoscale devices

## Abstract

The era of Big Data requires nanophotonic chips to have large information processing capacity. Multiple frequency on-chip nanophotonic devices are highly desirable for density integration, but such devices are more susceptible to structural imperfection because of their nano-scale. Topological photonics provides a robust platform for next-generation nanophotonic chips. Here we give an experimental report of an on-chip nanophotonic topological rainbow realized by employing a translational deformation freedom as a synthetic dimension. The topological rainbow can separate, slow, and trap topological photonic states of different frequencies into different positions. A homemade scattering scanning near-field optical microscope with high resolution is introduced to directly measure the topological rainbow effect of the silicon-based photonic chip. The topological rainbow based on synthetic dimension have no restrictions for optical lattice types, symmetries, materials, wavelength band, and is easy for on-chip integration. This work builds a bridge between silicon chip technologies and topological photonics.

## Abstract (web summary)

Here the authors provide the experimental observation of a topological rainbow in a silicon-based nanophotonic chip. The system is robust against disorders allows to separate and trap topological photonic states of different wavelength into different positions.

## Introduction

In the past few years, topological photonics have made great progress from physical concept to new applications^1–6^. Photonic devices become more robust against disorder and immune to scattering due to topological protection^7–9^. However, topological nanophotonic devices^9–16^ are difficult to realize because of complexity of fabrication, challenges of measurement in the nano-scale, and the fact that magnetic response is inherently weak for natural materials in the visible and near infra-red range. The introduction of synthetic dimensions has brought opportunities and insight into topological photonics where the topology goes beyond the geometric dimensions^17–26^. Synthetic dimension provides a new degree of freedom which enables us to construct all dielectric on-chip topological nanophotonic components, breaking the restrictions of magnetic materials^27^. In topological photonics research, much attention has been paid to engineer the bulk band dispersion to realize topological states in order to achieve unidirectional propagation, topological laser, high-order topology, etc.^7,28–30^. Multi-frequency (or multi-wavelength) devices are essential components of nanophotonic chips for large information capacity applications, among which, topological rainbow, as a basic multi-wavelength topological photonic device, can separate and distribute different wavelengths of topological photonic states into different positions; but related topological devices have not yet been fully explored. Moreover, no effective method has been found to give direct measurement of topological photonic devices with multiple wavelengths at the nanoscale to date. These challenges have restricted the development and applications of topological rainbow and various topological nanophotonic devices, including topological router, topological temporary storage, and many other on-chip integrated topological nanophotonic devices.

In this work, we have constructed a topological rainbow device based on a synthetic dimension, which provides a method that is universally applicable for all optical lattice types, symmetries, materials, dimensions, and wavelength range. Topological rainbow can separate and distribute different wavelengths of topological photonic states into different positions, and the light can be slowed and trapped through controlling the group velocities of the topological photonic states^31–33^. Here, the topological photonic states are realized through constructing topological “Chern insulators” without the need of a magnetic field. The on-chip silicon-based topological photonic crystal (PC) was successfully fabricated with a small size of only 4.5 μm × 22 μm. A homemade scattering scanning near-field optical microscope (*s*-SNOM) system with high enough resolution was set up to directly measure the electric field of the topological rainbow. Topological states of different wavelengths from 1540 to 1630 nm around the optical communication range have been separated, slowed, and trapped into different positions along the interface of PCs, which forms a nano-scale topological rainbow on the silicon chip. The group velocities can be decreased to zero in different interface positions of PCs, where the slow-light effect of the topological states is realized. The topological nanophotonic device based on a synthetic dimension is robust against disorder and can be implemented with purely dielectric materials and is hence compatible with photonic chip integration.

## Results

### Construction and design of the topological rainbow

**Fig. 1:**
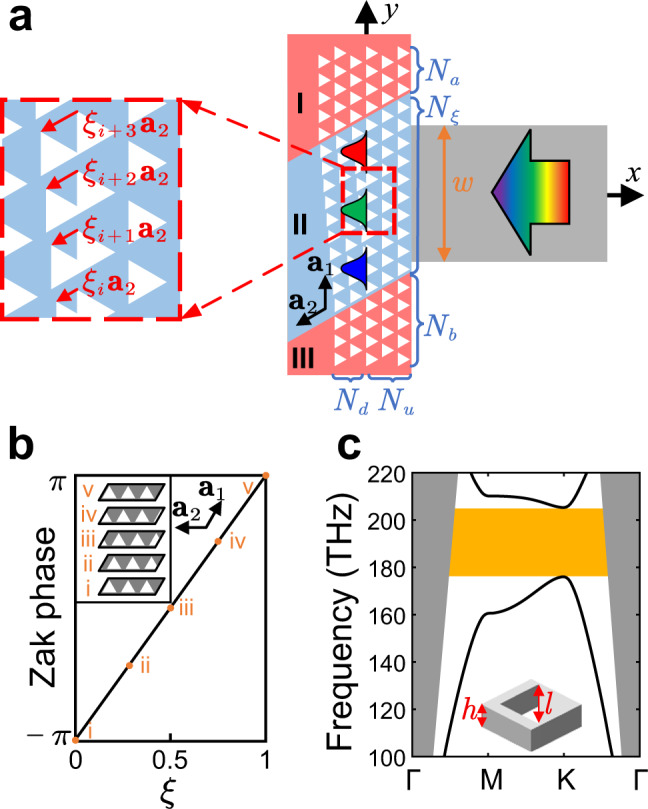
Geometric structure, bulk bands, and Zak phase. **a** Schematic diagram of the topological rainbow configuration. **a**_1_, **a**_2_ denote the lattice vectors. The red and blue regions denote the barrier and dispersing regions, respectively. The displacement vector of *i*th layer is denoted by *ξ*_*i*_**a**_2_. *N*_*a*_, *N*_*ξ*_, *N*_*b*_ denote the number of layers of regions I, III along **a**_1_ direction, and *N*_*u*_, *N*_*d*_ denote the number of layers of undeformed region and the deformed region in region II. Light is incident from the dielectric waveguide with a width *w* = 8 μm. **b** Evolution of Zak phases with parameter *ξ*. The inset shows the geometric structure for different *ξ*s. **c** The TE-like bands of triangular hole structure with side length *l* = 0.75*a* and thickness *h* = 220 nm. The geometry of the unit cell is shown in the inset.

In this section, the basic ideas about the construction of the topological rainbow are given in detail. The geometric structure of the topological rainbow is shown in Fig. 1a, which consists of three regions. The blue region (Region II) is the dispersing region, which separates and distributes different wavelengths of topological photonic states into different positions (and hence called “rainbow”) due to the non-trivial topology in synthetic dimension. The red regions (Regions I and III) are barrier regions, which provide a bandgap to prevent the leakage of light. The purpose of adding blue or red color is to display different regions visually. The barrier regions consist of ordinary PCs with a complete bandgap, and the lattice vectors are denoted by **a**_1_, **a**_2_. The dispersing region is constructed by an undeformed PC on one side of the *y*-axis and a graded translationally deformed PC on the other, where the vector of the displacement is along lattice vector **a**_2_. The ratio between the displacement of the *i*th layer and the lattice vector is defined as the translational parameter *ξ*_*i*_, as is shown in the inset of Fig. 1a. The parameters *N*_*a*_, *N*_*ξ*_, *N*_*b*_ denote the number of layers of regions I, II, III along **a**_1_ direction, and *N*_*u*_, *N*_*d*_ denotes the number of layers of undeformed region and the deformed region in region II, respectively. External light signal is incident from a waveguide, and the frequency range lies within the bulk bandgap of the PC.

The functionality of the dispersing region results from the non-trivial topology in the lattice translational deformation dimension. We find two ways to achieve nontrivial topology using a synthetic dimension in PCs, one is based directly translational PC, and the other is based on twisted PC (see Supplementary Fig. 1). The details are illustrated in the Supplementary Materials Sec. 1. We take the first way of constructing synthetic dimension for the following experimental verification. Here we consider a uniformly deformed PC along **a**_2_ direction, and the ratio between the displacement and the lattice constant is denoted by *ξ*. The Bloch wavevector is decomposed by using the basis of reciprocal lattice vectors **k** = *k*_1_**b**_1_ + *k*_2_**b**_2_, where **b**_1_ and **b**_2_ satisfy ${{\mathbf{a}}}_{i}\cdot{{\mathbf{b}}}_{j}=2\pi\delta_{ij}$ . Here, the Bloch states and the wavevectors are defined either in the deformed or in the undeformed domains alone, where the periodicity is preserved so that the wavevectors are well defined. The parameters (*k*_1_, *k*_2_, *ξ*) form a three-dimensional synthetic space. With the value of *k*_1_ fixed, the Zak phase^34^ of the *k*_2_ dimension is defined as Eq. 1,

1 $${\theta }_{n}^{({{{{{\rm{Zak}}}}}})}({k}_{1},\xi )=\int_{-1/2}^{1/2}\langle {u}_{n}({k}_{1},{k}_{2},\xi )|{{{{{\rm{i}}}}}}\frac{\partial }{\partial {k}_{2}}|{u}_{n}({k}_{1},{k}_{2},\xi )\rangle {{{{{\rm{d}}}}}}{k}_{2},$$

where ${\theta }_{n}^{({{{{{\rm{Zak}}}}}})}({k}_{1},\xi )$ denotes the Zak phase corresponding to parameter (*k*_1_, *ξ*) and band *n*. As the displacement parameter *ξ* goes from −1/2 to 1/2, the PC goes back to itself and the Zak phase evolution along the *ξ* path can be used to define a Chern number. In the three-dimensional synthetic space, when *k*_1_ is fixed, and the two-dimensional subspace (*k*_2_, *ξ*) chosen, the Chern number is related to the evolution of Zak phase when *ξ* changes by a period, as is shown by Eq. 2^35^,

2 $${C}_{n}({k}_{1})=\frac{1}{2\pi }\int_{-1/2}^{1/2}{\partial }_{\xi }{\theta }_{n}^{({{{{{\rm{Zak}}}}}})}({k}_{1},\xi ){{{{{\rm{d}}}}}}\xi$$

where *C*_*n*_(*k*_1_) is the Chern number of the band *n* and parameter *k*_1_. Equation 2 means that the Chern number of the subspace (*k*_2_, *ξ*) specified by a fixed value of *k*_1_ equals the winding number of the Zak phase when *ξ* changes by a period. The Wannier center will change when *ξ* changes by a period, so that the Zak phase changes by 2*π*, as is shown in Fig. 1b, and the Chern number of the 2D synthetic subspace (*k*_2_, *ξ*) is 1. For all values of *k*_1_, the results are the same. More detailed discussion of the non-trivial topology of synthetic dimension is shown in the Supplementary Materials Sec. 1.

According to the bulk-edge correspondence^36,37^, the net number of interface states going in one direction equals the difference of the Chern numbers of systems on either side of the interface. For synthetic dimensions, the “direction” of the interface dispersion is also defined in synthetic space, that is, the direction along which eigenfrequencies changes when the synthetic parameter changes by a period. Because the non-trivial topology utilized here lies in the synthetic space (*k*_2_, *ξ*), the deformed and the undeformed PC act as the topological and trivial structures, and are joined together in the direction of **a**_2_. The non-trivial “topology” ensures that the eigenfrequencies of the interface states change with *ξ* and cross the bulk bandgap when *ξ* changes by a period. Therefore, the interface states with different frequencies are separated when *ξ* is modulated spatially, which realizes the functionality of a rainbow whose dispersion relation is protected by the topology in the synthetic dimension^27,36^.

**Fig. 2:**
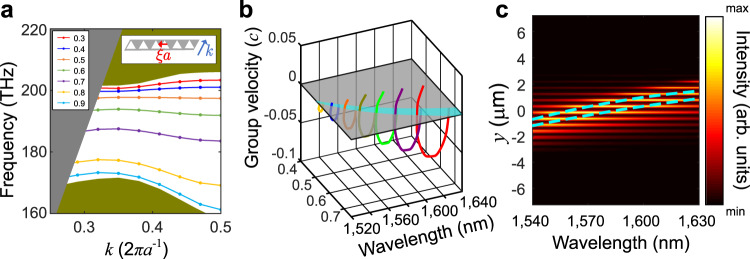
Bands and group velocities of the interface states. **a** The interface bands of TE-like modes. The grey domain denotes the light cone, and the greyish-green domain denotes the projected bulk bands. The inset shows the supercell of the spliced structure, where the grey part is silicon film and the white triangles are holes. The translational parameter (red arrow) and the wavevector (blue arrow) along the interface are marked. **b** Group velocity as a function of parameter *ξ* and wavelength. The colored lines are group velocity lines for *ξ* = 0.52, 0.55, 0.58, 0.61, 0.64, 0.67, and 0.70, respectively. The zero-group-velocity plane is marked by the grey plane. The cyan region denotes the projection of group velocity lines. **c** Calculated results of interface intensity for different incident wavelengths. The green dashed lines mark the boundaries of the projected interface bands, where the dimension *ξ* is substituted by the coordinate *y*.

Because the topology discussed above can exist as long as the bulk PC has a bandgap, the geometric structure and the material of the rainbow can be designed to satisfy the target frequency. In order to realize rainbow separation of light in the range from 1540 to 1630 nm, which is around the optical communication range and is also our tunable laser source range, we design a PC structure using all dielectric materials of silicon-on-insulator (SOI) whose bandgap covers the target range. The geometric structure and the band dispersions are shown in Fig. 1c. The lattice constant is *a* = 485 nm, the thickness of the silicon slab is *h* = 220 nm, and the side length of the triangle is *l* = 0.75*a*. Figure 1c shows the bulk bands of transverse electric (TE)-like modes, where the orange stripe marks the photonic bandgap from 172.4 to 201.1 THz (from 1491 to 1739 nm in wavelength), and the grey region denotes the light cone. Under the bandgap marked by the orange stripe, there is one isolated band with Chern number 1 in the synthetic dimension, and therefore only one interface band is expected at the interface of spliced structure. Figure 2a shows the calculated results of the dispersion of interface states for different values of the synthetic dimension *ξ*. The inset shows the geometric structure of the supercell, and the translational deformation is marked by the red vector and the wave vector is marked by the blue vector. The grey and greyish-green regions denote the light cone and the projected bulk bands, respectively. The colored curves are the bands of interface states corresponding to different *ξ*. As is shown in Fig. 2a, when *ξ* changes by a period, the frequency of the interface states for given wavevector *k* varies across the bandgap. It should be pointed out that the topological rainbow shows advantage to conventional designs, because as long as a band-gapped photonic crystal is constructed, the topological rainbow will definitely exist due to the non-trivial topology.

The group velocities of interface states can also be controlled by modulating of dispersion. In Fig. 2b, the calculated results of group velocities are shown in the space of parameter *ξ* and wavelength (see Supplementary Materials Sec. 2 for the detail of the calculation method). Because of the time-reversal symmetry, only the group velocities of states with the positive wavevectors are shown. The zero-group-velocity plane is marked in grey, and the area bounded by the states with zero group velocity is marked in cyan. Because the zero-group-velocity points corresponds to wavelength extrema, the cyan region is also the projection of the interface dispersion bands on the *ξ* dimension. We call the region “projected interface bands” in the following text. Because of the topology in *ξ* dimension, when *ξ* increases, the wavelengths of the states with zero group velocity increase with *ξ*, which can be employed to stop or separate light with different frequencies. It demonstrates that the notion of a synthetic dimension provides an effective way to tune group velocity of topological photonic states. Therefore, topological photonic states with different frequencies can be slowed and used for information temporary storage. Furthermore, by coupling the interface modes to output waveguides, a topological router can be constructed, as is shown in Supplementary Fig. 2. A detailed discussion is available in the Sec. 3 of Supplementary Materials.

We calculate the light intensity distributions of the topological rainbow by using the finite difference time domain (FDTD) method. The intensity distributions at the interface of un-deformed PC and deformed PC are shown in Fig. 2c, and the boundaries of the projected interface bands are marked by cyan dashed lines, where the parameter *ξ* is substituted by the corresponding coordinate *y*. The maximum intensity of different wavelengths is trapped in different positions of the interface. As the propagating interface modes are only supported in the projected interface bands, light mainly concentrates in the region of projected interface bands, and the intensity decreases exponentially outside that domain. A time-domain simulation is shown in the Supplementary Videos S1 and S2 of the Supplementary Materials to demonstrate that light is trapped at the interface.

It should be pointed out that the intensity distributions of modes with different wavelengths can be well separated by using other materials or structures, and an example is given in Supplementary Fig. 3 in the Supplementary Materials. A topological rainbow with highly localized interface states can be constructed for at least 7 distinguishable wavelengths (1200, 1240, 1277, 1323, 1459, 1606, and 1772 nm) within the range of bulk bandgap (from 1185 to 1811 nm). The corresponding *y* coordinates of the point with maximal intensity are −1953, −1619, −1279, −953, 400, 1699, and 2606 nm, respectively. The displacement rate is Δ*y*/Δ*λ* = 7.97, which is the ratio of the total displacement of the positions with maximal intensity over the working bandwidth, and different modes are separated completely in space because the modes are highly localized.

Moreover, higher-dimensional spatial topological rainbow trapping can also be constructed beyond 2D planar nano-structures based on synthetic dimensions. We give an example of a 3D topological rainbow based on a dielectric PC in a diamond lattice. Similar to the 2D planar cases, the 3D topological rainbow also shows rainbow effect, and the difference lies in that the 3D topological rainbow supports dispersed conductive channels rather than dispersed localized states. A detailed discussion is in Supplementary Materials Sec. 5 (Supplementary Figs. 4, 5, and 6). This also demonstrates the notion of synthetic dimension is applicable for the study of higher-dimensional and more complex physical systems.

### Experimental realization of topological rainbow

**Fig. 3:**
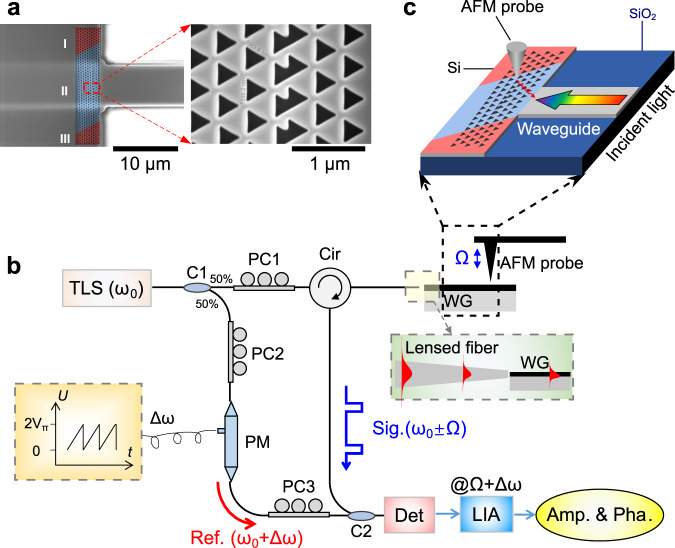
SEM image and the schematic diagram of the electric field detection. **a** SEM images of the sample. Barrier regions and the dispersing region are colored by pseudo red and blue respectively to display different regions more clearly visually. The width of the pattern for regions (I, II, and III) is 4.5 μm in the *x-*direction, the length is 22 μm in the *y-*direction, and the length of region II for rainbow is 16 μm. **b** Experimental setup of all-fiber reflection-based *s*-SNOM. TLS: tunable laser source; C1, C2: in-line 50:50 directional couplers; PC1, PC2, PC3: polarization controller; Cir: circulator; WG: waveguide under test; PM: LiNbO_3_ phase modulator; Vπ: half wave voltage of PM; Det: InGaAs photodetector; LIA: digital lock-in amplifier. **c** Amplified schematic diagram of the electric field detection based on the *s*-SNOM system in (**b**).

The topological rainbow proposed here can be verified experimentally in the optical frequency range by silicon-based technologies. The samples are fabricated on a common SOI chip comprising a 220 nm-thick silicon layer and a 2 μm-thick SiO_2_ layer. The fabrication methods and scanning electron microscope (SEM) images of samples in a large scale are shown in Supplementary Fig. 7 in the Supplementary Materials. The geometric parameters for the structure are *N*_*a*_ = 6, *N*_*b*_ = 9, *N*_*ξ*_ = 30, *N*_*u*_ = 3, *N*_*d*_ = 6, matching the geometry of the input waveguide. The parameter *N*_*ξ*_ = 30 determines the variation rate of the synthetic parameter *ξ* along the interface. A detailed discussion is in the Sec. 7 of the Supplementary Materials, and the calculated results of different *N*_*ξ*_ are shown in Supplementary Fig. 8. The central axis of the whole structure is aligned to the waveguide. The SEM images are shown in Fig. 3a, where the barrier regions and dispersing region are colored with pseudo red and green color respectively to display the pattern clearly.

To accurately characterize the performance of such an on-chip topological rainbow device, it requires a SNOM system that has the simultaneous capability of sub-structure spatial resolution, high optical collection efficiency, and scanning repeatability. There are two typical ways for measurement based on SNOM. One is to employ tapered fibers with an aperture, which is also called collected SNOM with a resolution of about 100 nm, depending on the diameter of the aperture. The other is to employ atomic force microscope (AFM) tips that offer a higher resolution (less than 20 nm) depending on the diameter of the tip, which is also called scattering SNOM (*s*-SNOM)^38^. Nearly all previous nanophotonic topological functionalities can also be conveniently identified in the far field, but for the on-chip ultra-compact topological rainbow device, the *s*-SNOM system may be the unique technique to give a direct measurement with high enough resolution for both topography and optical field. In our work, different from aperture-fiber SNOM system used in recent works^16,39^, we adopt a homemade reflection-based *s*-SNOM system with a higher spatial resolution of ~20 nm, which can directly measure each lattice topography and its corresponding optical intensity simultaneously with high enough resolution to demonstrate each nanophotonic lattice.

Figure 3b shows the home-made reflection-based scattering scanning near-field optical microscope (*s*-SNOM), which consists of an AFM module (NT-MDT Spectrum Instruments, NTEGRA Solaris) and a fiber Mach–Zehnder interferometer (MZI) with heterodyne detection. The near-field microscopy is based on a cantilevered AFM probe as a near-field probe. In this work, the silicon AFM probe (Scansens GmbH, HA_NC) has a typical tip radius of ~10 nm and cone full angle of 30°, which ensures that the topography and the optical field can be acquired simultaneously with a spatial resolution of ~20 nm. Operating in the tapping mode, the AFM probe vibrates vertically at a fixed frequency of Ω ≈ 140 kHz with the amplitude of ~100 nm. The interaction of light with the AFM probe results in the conversion of near field to backward guided modes in the input waveguide, forming the modulated signal light. This reflection-based *s*-SNOM has high light collection efficiency because the waveguide can naturally play the role of a high-numerical-aperture lens, as is shown in Fig. 3c.

## Discussion

Our reflection-based *s*-SNOM system has several advantages. Firstly, the AFM tip can penetrate the nano-structures and couple with the light confined in the air holes, which can significantly increase the collection efficiency. Secondly, thanks to the high resolution, the topography, and the optical field can be acquired simultaneously, and the deviation of the scanning area in different scanning process can be corrected according to the topography image, providing an effective method to measure the surface intensity of topological nanophotonic device directly. Thirdly, the all-fiber setup with a single coupling point configuration can provide a convenient, high light collection efficiency, and low background noise platform^40^ for the characterization of on-chip structure. Based on the above characteristics, the *s*-SNOM makes the direct observation of on-chip nanophotonic topological rainbow with high resolution.

**Fig. 4:**
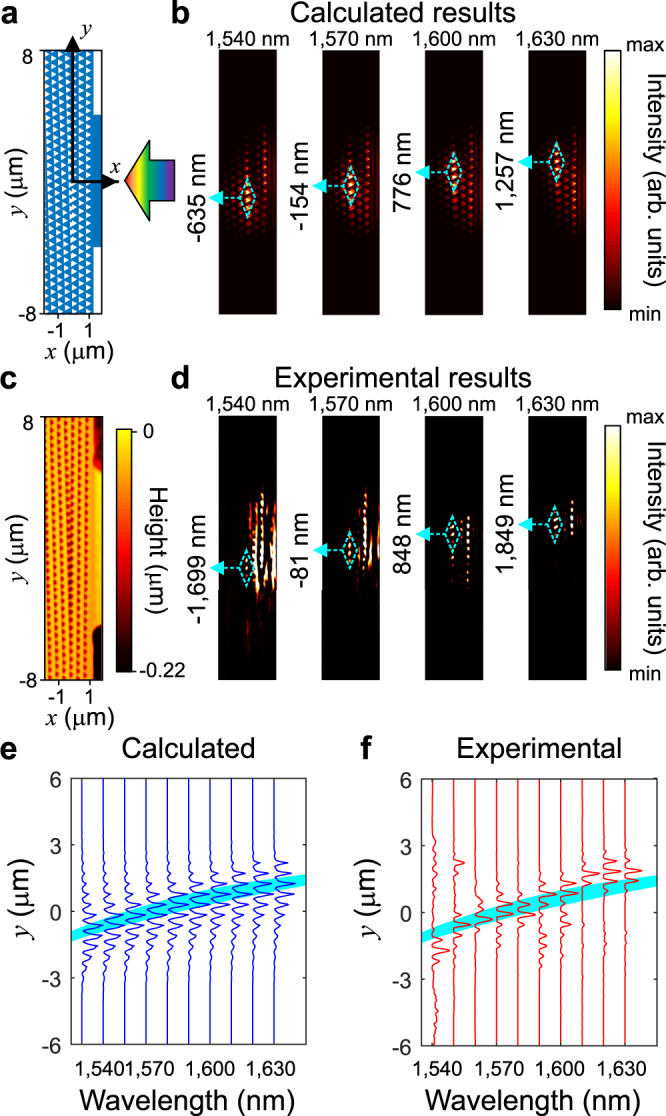
Calculated and experimental results of the topological rainbow. **a** The top view of the FDTD model, where coordinate axes are marked and light is incident from the waveguide. **b** The light intensity distributions (|E|^2^) of the calculated results for different wavelengths. **c** The topographic image of the sample. The color denotes the height of the surface of the sample. **d** The light intensity distributions of experimental results for different wavelengths. The comparison between interface intensity and projected bands for calculated (**e**) and experimental (**f**) results. In **b**, **d**, the position with maximal intensity is marked by the cyan dashed rhombuses, and the corresponding *y* coordinates are marked in the left. The wavelength of incident light is marked on the top of each figure.

Next, we show the experimental and calculated results of the topological nanophotonic rainbow. In order to characterize the light separation ability of the rainbow, the *y* coordinates of the points with maximal intensity along the interface are measured, and displacement rate Δ*y*/Δ*λ*, is defined to characterize the light separation ability of the rainbow device. For the calculated results, the top view of the calculated FDTD model is shown in Fig. 4a, where the blue part denotes the silicon wafer and the white part denotes the hole, and the light intensity distributions (|*E*|^2^) for four wavelengths are shown in Fig. 4b. For wavelengths 1540, 1570, 1600, and 1630 nm, the *y-*coordinates of the points with maximal intensity are −635, −154, 776, and 1257 nm, respectively, and the displacement rate is 21.02. For the experimental results, the topographic image of the sample and the surface intensity distributions are measured simultaneously by the *s*-SNOM system. Figure 4c is the topographic picture of the surface of sample. The color denotes the height of the sample surface. Figure 4d shows the measured intensity distributions for the four wavelengths. The positions with maximal electric intensity are marked by the cyan dashed rhombuses. The position of maximal intensity moves towards the positive direction of *y-*axis when wavelength increases. The *y* coordinates of the maximal electric intensity are −1699, −81, 848, and 1849 nm for the four wavelengths, respectively, and the displacement rate is 39.42. This relation is consistent with the topology in the lattice translational dimension. As is predicted by the dispersion bands, when wavelength increases (frequency decreases), the position of the interface state moves to the position with the increase of *ξ*.

Figure 4e, f shows the comparison between the interface intensity of the topological rainbow and the projected interface bands. Similar to Fig. 2d, the *ξ* dimension of the projected interface bands is substituted by *y*, and the interface intensity is averaged in *x* direction within a distance of $\sqrt{3}a/4$ from the interface. For the calculated results, the patterns of intensity distributions are the same as discussed previously, that the light concentrates in the projected interface bands region and decreases exponentially out of the region. For the experimental results, the light is also mainly concentrated in the projected interface bands region, and the states in the interface of rainbow moves when the wavelength changes. The positions of different rainbow states are related to the dispersion of topological edge states, though the intensity distributions of calculated and experimental results have slight differences. There are three main reasons that cause the differences. First, the AFM probe dose not only reflect the light in the rainbow device to the waveguide, but also scatters the light in the free space into the waveguide, which adds noise and causes the signal of stray light. Second, the waveguide is wide compared to the wavelength of incident light, which will support multi-mode propagation. The interference of different modes will influence the spatial distribution of rainbow states. Third, some evanescent modes within the light cone may be excited under continuous excitation during the measurement, and they may be collected by the AFM probe. Apart from the above triangular hole structure, the topological rainbow can also be constructed by other structures. In the Supplementary Materials, we also present the calculated and experimental topological rainbow for square air holes nano-structures in Supplementary Figs. 9 and 10.

In conclusion, we have experimentally realized on-chip topological rainbow nanophotonic devices by introducing a synthetic dimension to a dielectric photonic crystal. The silicon-based PC sample was successfully fabricated with an ultra-compact size of 4.5 μm × 22 μm. Topological states of different wavelengths from 1540 to 1630 nm around the optical communication range have been separated and trapped at different positions along the interface of the photonic crystals. The rainbow effect is directly demonstrated using a homemade *s*-SNOM system for different optical lattice nano-structures. It offers an effective way to implement on-chip nanophotonic rainbow devices enabled by topological ideas, using the notion of synthetic dimensions. This work provides a method for constructing topological states using dielectric materials without using magnetic field or magnetic materials in the optical frequency range. The topological rainbow effect can be used for topological slow light, topological router, and topological temporary storage, enabled by the fact that different frequencies of topological photonic states can have different group velocities with suitably chosen sample structural parameters. The work provides an avenue to the implementation of on-chip nanophotonic topological devices beyond the structure’s band topology, such as realizing non-zero Chern numbers in dielectric systems, and can stimulate the development of topological nanophotonic chips.

## Methods

### Theoretical calculations

The detailed derivation of the non-trivial topology in synthetic dimensions for arbitrary lattice type is shown in Supplementary Materials Sec. 1. The bands of the bulk states and the edge states are calculated by the finite element method, and the intensity distribution of the topological rainbow is calculated by the FDTD method. The numerical calculation method of the group velocity is shown in Supplementary Materials Sec. 2.

### Experimental measurements

The samples are mainly fabricated by a focused-ion-beam system. The details of the sample fabrication method are shown in Supplementary Materials Sec. 6. The sample is illuminated from a tunable laser (Agilent, 81940A), with the wavelength ranging from 1520 to 1630 nm and the line-width of <100 kHz. The continuous-wave laser light is separated to signal and reference arm through a fiber directional coupler. In the signal arm of the MZI, a lensed fiber (CXFIBER) launches the light (*ω*_0_) into the waveguide under tested and collects the modulated reflection light (*ω*_0_ ± Ω) produced by the AFM probe from the same facet. The three-paddle polarization controller ensures that the guided modes in the waveguide are quasi-TE polarization. In the reference arm of the MZI, the light is frequency-shifted by Δ*ω* = 30 kHz via an in-line LiNbO_3_ phase modulator (SWT Sci. & Tech.) linked with a saw-tooth waveform generator. The all-fiber characteristic brings about convenience (alignment free with single coupling point), compactness, and low background noise, which is suitable for the near-field imaging of on chip photonic circuits. Then, both the signal (*ω*_0_ ± Ω) and the reference (*ω*_0_ + Δ*ω*) light are combined and sent to an InGaAs amplified photodetector (Thorlabs, PDA10CS2). The photocurrent can accurately yield the amplitude and phase of the signal light through a lock-in amplifier (Zurich Instruments, HF2LI) at the demodulation frequency of Ω + Δ*ω*.

## Supplementary information

Supplementary Information

Time-domain calculation for 1,540 nm

Time-domain calculation for 1,580 nm

## Data Availability

All data and materials in the manuscript or the supplementary materials are available from Cuicui Lu.

## Code availability

All codes in the main text or the supplementary materials are available from Cuicui Lu.
