# Supplementary material for: On-chip nanophotonic topological rainbow
Source: Nat Commun. 2022 May 11;13:2586. doi: 10.1038/s41467-022-30276-w (PMC9095703; doi:10.1038/s41467-022-30276-w)
Supplement: Supplementary file 1 — Supplementary Information [file 41467_2022_30276_MOESM1_ESM.docx]

Supplementary Materials

**On-chip nanophotonic topological rainbow**

Cuicui Lu,^1,2^*†, Yi-Zhi Sun^3^†, Chenyang Wang^1^†, Hongyu Zhang^1^, Wen Zhao^1^, Xiaoyong Hu^4^*, Meng Xiao^5^, Wei Ding^3^*, Yong-Chun Liu^6^, C. T. Chan^7^

**Affiliations:**

^1^Key Laboratory of Advanced Optoelectronic Quantum Architecture and Measurements of Ministry of Education, Beijing Key Laboratory of Nanophotonics and Ultrafine Optoelectronic Systems, School of Physics, Beijing Institute of Technology; Beijing 100081, China.

^2^Collaborative Innovation Center of Light Manipulations and Applications, Shandong Normal University; Jinan 250358, China.

^3^Institute of Photonics Technology, Jinan University; Guangzhou 510632, China.

^4^State Key Laboratory for Mesoscopic Physics & Department of Physics, Collaborative Innovation Center of Quantum Matter & Frontiers Science Center for Nano-optoelectronics, Beijing Academy of Quantum Information Sciences, Peking University; Beijing 100081, China.

^5^Key Laboratory of Artificial Micro- and Nano-Structures of Ministry of Education, School of Physics and Technology, Wuhan University; Wuhan 430072, China.

^6^State Key Laboratory of Low-Dimensional Quantum Physics, Department of Physics, Frontier Science Center for Quantum Information, Tsinghua University; Beijing 100084, China.

^7^Department of Physics, The Hong Kong University of Science and Technology; Clear Water Bay, Kowloon, Hong Kong, China.

†These authors contributed equally to this work.

*Corresponding author. Email: [cuicuilu@bit.edu.cn](mailto:cuicuilu@bit.edu.cn); [xiaoyonghu@pku.edu.cn](mailto:xiaoyonghu@pku.edu.cn); [dingwei@jnu.edu.cn](mailto:dingwei@jnu.edu.cn).

**Contents**

[1. Nontrivial topology of synthetic dimension in photonic crystals 3](#_Toc95082203)

[1.1 Topology in direct translational photonic crystals 3](#_Toc95082204)

[1.2 Topology in twisted photonic crystals 5](#_Toc95082205)

[2. Calculation of group velocity of interface states 9](#_Toc95082206)

[3. Topological routers based on topological rainbow effect 13](#_Toc95082207)

[4. Topological rainbow without overlaps of intensity 15](#_Toc95082208)

[5. The topological rainbow in higher dimensions 19](#_Toc95082209)

[6. Sample fabrication methods and SEM images in a large scale 27](#_Toc95082210)

[7. Different variation rate of the synthetic parameter *ξ* 30](#_Toc95082211)

[8. Topological rainbow for square holes structures 34](#_Toc95082212)

[**References and Notes**](#_Toc95082213) 39

**Contents of Supplementary Videos**

Supplementary Video 1:

Time domain calculation of the topological rainbow for incident wavelength 1,540 nm. The white solid lines mark the edge of silicon wafer, the white dashed lines mark the interface between deformed and un-deformed PCs, and the boundaries of the dispersing and barrier regions. The cyan lines mark the boundaries of the projected dispersion bands at wavelength 1,540 nm.

**Supplementary Video 2:**

**Time domain calculation of the topological rainbow for incident wavelength 1,580 nm.** The white solid lines mark the edge of silicon wafer, the white dashed lines mark the interface between deformed and un-deformed PCs, and the boundaries of the dispersing and barrier regions. The cyan lines mark the boundaries of the projected dispersion bands at wavelength 1,580 nm.

1. Nontrivial topology of synthetic dimension in photonic crystals

There are two ways of constructing nontrivial topology of synthetic dimension. One is based on directional translational photonic crystals, which is also adopted as our experimental sample design since it is easier to fabricate. The second way to construct nontrivial topology of synthetic dimension is based on twisted photonic crystals.

1.1 Topology in direct translational photonic crystals

The photonic crystal lattice vectors are denoted by **a**_1_, **a**_2_. In order to demonstrate the concept of topological pumping, the concept of hybrid Wannier function is introduced. The hybrid Wannier function is defined as Supplementary Equation (1), which is constructed by the Fourier transformation of Bloch states in the direction **a**_2_, and keeping the Bloch form in the direction **a**_1_.

$\begin{matrix} |W_{n}\left(k_{1},R,\xi\right)\rangle= \int_{-1/2}^{1/2} e^{-i\mathbf{k}\cdot R\mathbf{a}_{2}}|\psi_{n}(k_{1},k_{2},\xi)\rangle dk_{2}, \\ = \int_{-1/2}^{1/2} e^{-2\pi ik_{2}R}|\psi_{n}(k_{1},k_{2},\xi)\rangle dk_{2}. \end{matrix}$ (1)

Here, Bloch states are normalized as $\langle\psi_{n}(k_{1},k_{2},\xi)|\psi_{n}(k'_{1},k'_{2},\xi)\rangle=\delta(k_{1}-k'_{1})\delta(k_{2}-k'_{2})$. Correspondingly, the Bloch states can also be expanded in the basis of hybrid Wannier functions as Supplementary Equation (2),

$|\psi_{n}\left(k_{1},k_{2},\xi\right)\rangle=\sum_{R_{2}} e^{2\pi ik_{2}R_{2}}|W_{n}\left(k_{1},R_{2},\xi\right)\rangle$ (2)

where the sum of *R*_2_ is over all integers.

Similar to one-dimensional lattices where Zak phases are related to the centers of Wannier functions ^1^ in multi-dimensional cases, Zak phases, defined as equation (1) in the main text, are also related to the center of hybrid Wannier functions. We decompose the position operator $\hat{\mathbf{r}}$ in the basis of lattice vectors **a**_1_, **a**_2_, as is shown in Supplementary Equation (3).

$\hat{\mathbf{r}}=\hat{x}_{1}\mathbf{a}_{1}+\hat{x}_{2}\mathbf{a}_{2}$ (3)

The operators $\hat{x}_{1}$, $\hat{x}_{2}$ are components of position operator under the basis of lattice vectors. By direct computation, the relation between Zak phase and hybrid Wannier function is Supplementary Equation (4),

$\begin{matrix} \theta_{n}^{(\text{Zak})}(k_{1},\xi)= \int_{-1/2}^{1/2} \langle u_{n}(k_{1},k_{2},\xi)|i\frac{\partial}{\partial k_{2}}|u_{n}(k_{1},k_{2},\xi)\rangle dk_{2} \\ = \int_{-1/2}^{1/2} \{\sum_{R} e^{-2\pi ik_{2}R}e^{2\pi ik_{2}x_{2}+ik_{1}x_{1}}\langle W_{n}(k_{1},R,\xi)|\}\times\\ \times i\frac{\partial}{\partial k_{2}}\{\sum_{R'} e^{2\pi ik_{2}R'}e^{-2\pi i(k_{2}x_{2}+ik_{1}x_{1})}|W_{n}(k_{1},R',\xi)\rangle\}dk_{2} \\ = \int_{-1/2}^{1/2} \{\sum_{R} e^{-2\pi ik_{2}R}e^{2\pi ik_{2}x_{2}+ik_{1}x_{1}}\langle W_{n}(k_{1},R,\xi)|\}\times\\ \times\{\sum_{R'} e^{2\pi ik_{2}R'}e^{-2\pi i(k_{2}x_{2}+ik_{1}x_{1})}i(-2\pi i\hat{x}_{2})|W_{n}(k_{1},R',\xi)\rangle\}dk_{2} \\ = 2\pi\langle W_{n}(k_{1},R,\xi)|\hat{x}_{2}|W_{n}(k_{1},R,\xi)\rangle, \mathrm{mod} 2\pi. \end{matrix}$ (4)

Supplementary Equation (4) shows that the Zak phase is proportional to the position of the hybrid Wannier center along **a**_2_ lattice vector. The nontrivial topology results from the topological pumping mechanism, which can be understood as follows. When the lattice is translational deformed along **a**_2_, all of the Bloch states are deformed with the same displacement, thus, according to Supplementary Equation (3), the hybrid Wannier function is translationally deformed. When *ξ* changes by a period, the hybrid Wannier function will also change by a period. According to Supplementary Equation (4), Zak phase will change by 2*π*. According to the relation between Zak phase and Chern number, as is shown in equation (2) in the main text, the Chern number *C_n_*(*k*_1_) equals 1. Because the discussion above is independent on the value of *n* and *k*_1_, the relation *C_n_*(*k*_1_) = 1 holds for all *n* and *k*_1_.

**1.2 Topology in twisted photonic crystals**

The second way to construct nontrivial topology of synthetic dimension is based twisted photonic crystals. The topological photonic interfaces can be formed by two 2D PC with the same structures but different twisted angles as shown in Supplementary Fig. 1a, in which the left section is the trivial PC (undeformed lattice) and the right one is the nontrivial topological PC (twisted lattice). The area of the structure depicted in Supplementary Fig. 1a is $8\sqrt{3}a\times15a$. The blue dot “o” is the origin of the y-axis and represents the center of rotation. The point *y* depicts the location where the air holes center intersects with the *y*-axis along the direction of the reverse extension line of **a**_2_. We introduce a parameter *y* to represent the distance between $y'$ with o, and *w* is the twisted angle. It should be noted that the topological photonic edge states manifest at this interface, and an interesting topological phenomenon can be found at the boundary due to the nontrivial topological PC which provides a new degree of freedom to modulate the topological photonic state propagating along with this interface. The topological rainbow trapping can be achieved because lights of different wavelengths through the interface of the propagation direction are localized at different spatial positions.

Physically, we can explain this as follows. For the right part of the twisted lattice, the parameter *y* and the Bloch wave vector **k** together form a 3D parametric space (*k*_1_**G**­_1_, *k*_2_**G**_2_, *y*). We define *ξ* as a displacement parameter in the **a**_2_ direction and it satisfies the relationship $\xi/\sin w=y/\sin120^{^{\circ}}$. When *ξ* changes by one lattice constant, *y* will change from 0 to $\sqrt{3}a/(2\sin w)$. When we fix *k*_1_, the eigenstates of this 2D subsystem $|\Psi_{n}(k_{2}\mathbf{G}_{2},y)\rangle$ are topologically nontrivial. For each y, the Zak phase of the n-th band is defined as

$$\theta_{n}^{(\text{Zak})}(k_{1},y)=\int_{-1/2}^{1/2} dk_{2}\langle u_{n}(k_{1},k_{2},y)|i\frac{\partial}{\partial k_{2}}|u_{n}(k_{1},k_{2},y)\rangle$$

Where $|u_{n}(\mathbf{k},y)\rangle=e^{-i\mathbf{k}\cdot\widehat{\mathbf{r}}}|\Psi_{n}(\mathbf{k},y)\rangle$The topological property is reflected by the Chern numbers, which are the winding numbers of the one-dimensional Zak phase as *y* changes by one period.

$$C_{n}(k_{1})=\frac{1}{2\pi}\int_{-\frac{\sqrt{3}a}{4\sin w}}^{\frac{\sqrt{3}a}{4\sin w}}\frac{\partial}{\partial y}\theta_{n}^{(\text{Zak})}(k_{1},y)dy$$

As the parameter *ξ* is defined by moving the undeformed system along the **a**_2_ direction, the Bloch states of the translated system and the undeformed system satisfy

$$\Psi_{n,\mathbf{k},y}(\mathbf{r})=e^{i\chi(\mathbf{k},\xi)}\Psi_{n,\mathbf{k},0}(\mathbf{r}-\xi\mathbf{a}_{2})=e^{i\chi(\mathbf{k},2y\sin w/\sqrt{3})}\Psi_{n,\mathbf{k},0}(\mathbf{r}-\frac{2y\sin w}{\sqrt{3}}\mathbf{a}_{2})$$

The phase *χ*(**k**, *y*) is an arbitrary phase factor, and satisfies the relation

$$e^{i\chi(\mathbf{k},y)}=e^{i\chi(\mathbf{k}+\mathbf{G}_{i},y)}=e^{i\chi(\mathbf{k},y+\frac{\sqrt{3}a}{2\sin w})}$$

For a fixed *k*_1_, as we gradually change *y* from 0 to $\sqrt{3}a/(2\sin w)$, the Zak phase for each band will change by 2π and the Chern number is 1. Subsequently, by splicing together the normal structure and the twisted structure, a series of interface states can be constructed successively when *y* changes from 0 to $\sqrt{3}a/(2\sin w)$, i. e. different frequencies of topological photonic states are trapped into different positions to form a topological rainbow.

Supplementary Fig. 1b and Supplementary Fig. 1c show $\left|\mathbf{E}\right|^{2}$ field distributions of TM mode at $w=8^{^{\circ}}$ and TE mode at $w=13^{^{\circ}}$, respectively. In such a topological structure, the incident plane-wave at a certain frequency will be slowed down when propagating forward at this spatial position and the wave eventually “stopped” in principle, forming a topologically protected and robust against backscattering state. Evidently, light with different frequencies will stop at different spatial positions along the direction of propagation, rainbow trapping would occur. Furthermore, rainbow trapping was achieved in both TE and TM mode at the same range of band gap in this structure.


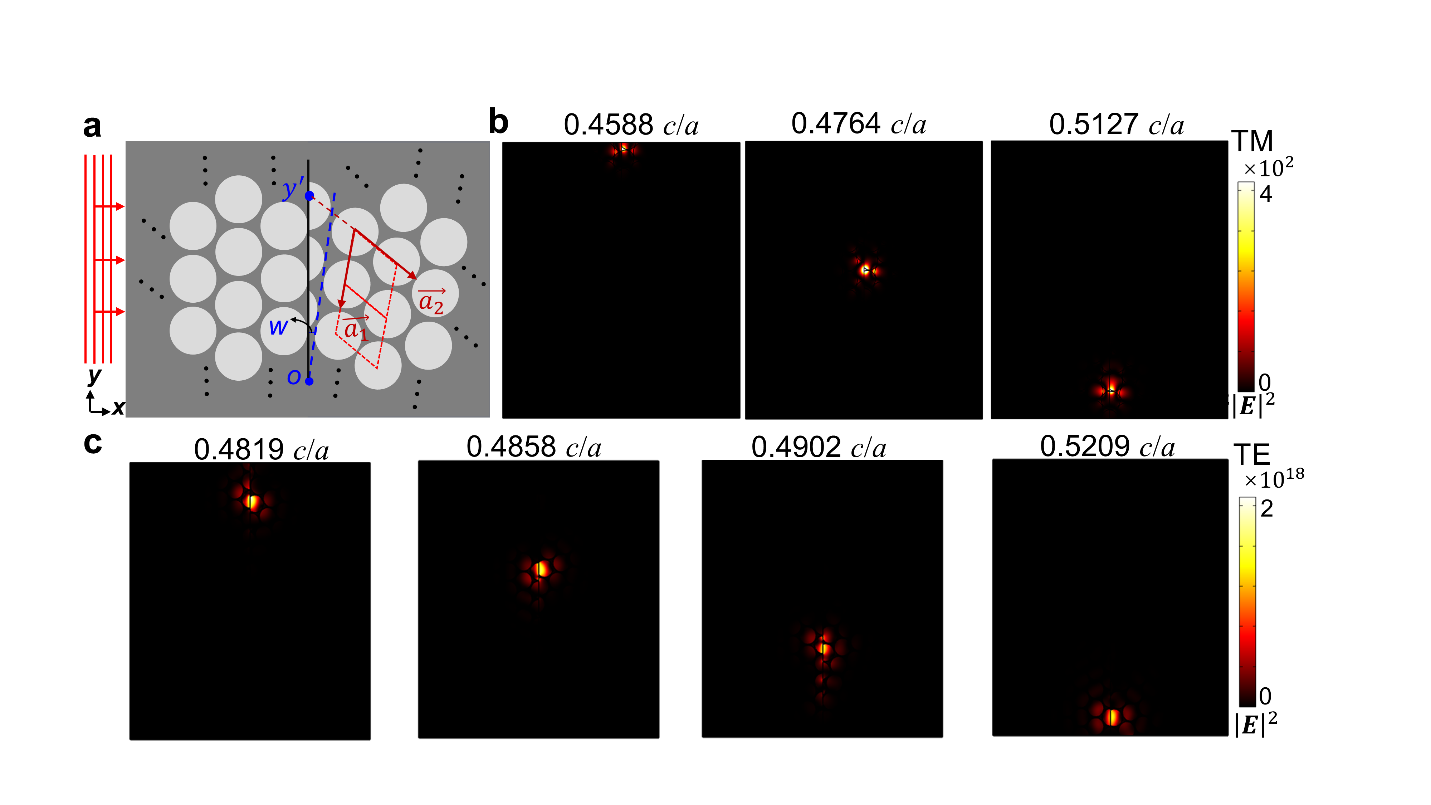


**Supplementary Fig. 1. Topological rainbow trapping effect at the interface of the twisted PC. a**, Schematic diagram of the proposed 2D topological PC, which is excited by plane wave broadband source from left to right along the x-direction. The PC structure comprises triangular lattices of air holes of radius $r=0.48a$ embedded in a dielectric slab of $n=3.45$, where *a* ($a=420/\sqrt{3}$nm) is the lattice constant. The solid black line illustrates the interface between nontrivial topological PC (twisted lattice) and trivial PC (normal lattice). **b**,**c**, The $\left|\mathbf{E}\right|^{2}$ field distributions of the TM mode at $w=8^{^{\circ}}$ and the TE mode at $w=13^{^{\circ}}$ respectively, which show obvious topological rainbow trapping effect.

1. Calculation of group velocity of interface states

The group velocities of the interface states are defined as the partial derivative of the eigen angular frequency *ω* over the wavevector *k* in the interface bands. For numerical calculations, the derivatives can be substituted by discrete finite differences, if the difference between two adjacent points in the discretized Brillouin zone is small enough. However, the calculation of eigenfrequencies of interface states in slab photonic crystals is time-consuming, making it hard to get the eigenfrequencies of a dense discretized Brillouin zone. Therefore, instead of substituting derivatives by finite differences, we use a photonic analog of Feynman-Hellmann theorem ^2^, that the derivatives of eigenfrequencies are calculated by the expectation value of the derivatives of Hamiltonian. Through this method, the eigenfrequency, the eigenstate and the group velocity of a certain mode can be simultaneously calculated in one eigenfrequency calculation.

We use the scheme proposed in Ref. ^3^ to derive the photonic analog of Feynman-Hellmann theorem. First, the frequency domain Maxwell equations can be rewritten in a Schrodinger equation form, as is shown in Supplementary Equation (9),

$\left[\begin{matrix} 0 & i\nabla\times\\ -i\nabla\times& 0 \end{matrix}\right]\left[\begin{matrix} \mathbf{E}(\mathbf{r}) \\ \mathbf{H}(\mathbf{r}) \end{matrix}\right]=\omega\left[\begin{matrix} \varepsilon(\mathbf{r}) & 0 \\ 0 & \mu(\mathbf{r}) \end{matrix}\right]\left[\begin{matrix} \mathbf{E}(\mathbf{r}) \\ \mathbf{H}(\mathbf{r}) \end{matrix}\right],$ (9)

where $\nabla\times$ denotes the curl operator, *ε*(**r**) and *μ*(**r**) are the permittivity and permeability tensors, and (**E**(**r**), **H**(**r**)) is the six-vector consisting of the complex amplitudes of electric and magnetic field. For photonic crystals, the complex amplitudes can be written in the Bloch form as Supplementary Equation (10),

$\left[\begin{matrix} \mathbf{E}\left(\mathbf{r}\right) \\ \mathbf{H}\left(\mathbf{r}\right) \end{matrix}\right]=e^{i\mathbf{k}\cdot\mathbf{r}}\mathbf{u}_{\mathbf{k}}\left(\mathbf{r}\right),$ (10)

where **u_k_**(**r**) is a periodic function with the same period of the photonic crystal, **k** is the Bloch wavevector. The eigen equation of **u_k_**(**r**) is derived by substituting Supplementary Equation (10) into Supplementary Equation (9), which is Supplementary Equation (11),

$\left[\begin{matrix} 0 & i(\nabla+i\mathbf{k})\times\\ -i(\nabla+i\mathbf{k})\times& 0 \end{matrix}\right]\mathbf{u}_{\mathbf{k}}(\mathbf{r})=\omega\left[\begin{matrix} \varepsilon(\mathbf{r}) & 0 \\ 0 & \mu(\mathbf{r}) \end{matrix}\right]\mathbf{u}_{\mathbf{k}}(\mathbf{r}),$ (11)

where the operator **k**× denotes the cross product of **k** and the three-vector components of **u_k_**(**r**). Next, the six-vector **u_k_**(**r**) is substituted by $\mathbf{w}_{\mathbf{k}}\left(\mathbf{r}\right)=\left[\begin{matrix} \varepsilon\left(\mathbf{r}\right) & 0 \\ 0 & \mu\left(\mathbf{r}\right) \end{matrix}\right]^{1/2}\mathbf{u}_{\mathbf{k}}\left(\mathbf{r}\right)$. Substituting **u_k_**(**r**) by **w_k_**(**r**) in Supplementary Equation (11), the classical wave analog of Schrodinger equation is derived as Supplementary Equation (12).

$\left[\begin{matrix} \varepsilon(\mathbf{r}) & 0 \\ 0 & \mu(\mathbf{r}) \end{matrix}\right]^{-1/2}\left[\begin{matrix} 0 & i(\nabla+i\mathbf{k})\times\\ -i(\nabla+i\mathbf{k})\times& 0 \end{matrix}\right]\left[\begin{matrix} \varepsilon(\mathbf{r}) & 0 \\ 0 & \mu(\mathbf{r}) \end{matrix}\right]^{-1/2}\mathbf{w}_{\mathbf{k}}(\mathbf{r})=\omega\mathbf{w}_{\mathbf{k}}(\mathbf{r}).$ (12)

In Supplementary Equation (12), the classical analog of Hamiltonian is shown as Supplementary Equation (13),

$\hat{H}=\left[\begin{matrix} \varepsilon(\mathbf{r}) & 0 \\ 0 & \mu(\mathbf{r}) \end{matrix}\right]^{-1/2}\left[\begin{matrix} 0 & i(\nabla+i\mathbf{k})\times\\ -i(\nabla+i\mathbf{k})\times& 0 \end{matrix}\right]\left[\begin{matrix} \varepsilon(\mathbf{r}) & 0 \\ 0 & \mu(\mathbf{r}) \end{matrix}\right]^{-1/2},$ (13)

and the analogs of wave function and eigen energy are respectively the six-vector field **w_k_**(**r**) and *ω*. Similar to the wave functions, the inner product of two six-vector fields **w**^(1)^(**r**), **w**^(2)^(**r**) can be defined as Supplementary Equation (14).

$\langle\mathbf{w}_{\mathbf{k}}^{(1)}|\mathbf{w}_{\mathbf{k}}^{(2)}\rangle=\iiint\left[\mathbf{w}^{(1)}(\mathbf{r})\right]^{\dagger}\mathbf{w}^{(2)}(\mathbf{r})d^{3}\mathbf{r}$ (14)

We introduce Dirac brakets $\langle\mathbf{w}|$ and $|\mathbf{w}\rangle$ to denote the normalized modes, which satisfy $\langle\mathbf{w}|\mathbf{w}\rangle=1$.

By establishing the analog between classical electromagnetic waves and quantum wave functions, the Feynman-Hellmann theorem can also be generalized to classical systems. Consider the derivative of eigen angular frequency over a parameter *α*, as is shown in Supplementary Equation (15), the derivative equals the expectation value of $\partial\hat{H}/\partial\alpha$.

$\begin{matrix} \frac{\partial\omega}{\partial\alpha}= \frac{\partial}{\partial\alpha}\langle\mathbf{w}(\alpha)|\hat{H}|\mathbf{w}(\alpha)\rangle, \\ = \left\{\frac{\partial}{\partial\alpha}\langle\mathbf{w}(\alpha)|\right\}\hat{H}|\mathbf{w}(\alpha)\rangle+\langle\mathbf{w}(\alpha)|\frac{\partial\hat{H}}{\partial\alpha}|\mathbf{w}(\alpha)\rangle+\langle\mathbf{w}(\alpha)|\hat{H}\left\{\frac{\partial}{\partial\alpha}|\mathbf{w}(\alpha)\rangle\right\}, \\ = \omega\frac{\partial}{\partial\alpha}\left\{\langle\mathbf{w}(\alpha)|\mathbf{w}(\alpha)\rangle\right\}+\langle\mathbf{w}(\alpha)|\frac{\partial\hat{H}}{\partial\alpha}|\mathbf{w}(\alpha)\rangle, \\ = \langle\mathbf{w}(\alpha)|\frac{\partial\hat{H}}{\partial\alpha}|\mathbf{w}(\alpha)\rangle, \end{matrix}$ (15)

where $|\mathbf{w}(\alpha)\rangle$ is the eigenstate with parameter *α*.

For interface states of photonic crystals, the Bloch wavevectors can be decomposed in the basis of reciprocal lattice vectors, which is **k** = *k***n**, -*π*/*a* < *k* < *π*/*a*, where the vector **k** is the Bloch wavevector, the scalar *k* is the magnitude of **k**, and **n** is the unit vector along the interface. Therefore, the derivative of Hamiltonian Supplementary Equation (13) over *k* is Supplementary Equation (16).

$\begin{matrix} v_{g}= \frac{\partial\omega}{\partial k}, \\ = \langle\mathbf{w}(k)|\frac{\partial\hat{H}}{\partial k}|\mathbf{w}(k)\rangle, \\ = \langle\mathbf{u}(k)|\left[\begin{matrix} 0 & -\mathbf{n}\times\\ \mathbf{n}\times& 0 \end{matrix}\right]|\mathbf{u}(k)\rangle, \\ = \iiint\left[\begin{matrix} \mathbf{E}^{\dagger}(\mathbf{r}) & \mathbf{H}^{\dagger}(\mathbf{r}) \end{matrix}\right]\left[\begin{matrix} 0 & -\mathbf{n}\times\\ \mathbf{n}\times& 0 \end{matrix}\right]\left[\begin{matrix} \mathbf{E}(\mathbf{r}) \\ \mathbf{H}(\mathbf{r}) \end{matrix}\right]d^{3}\mathbf{r}, \\ = \mathbf{n}\cdot\iiint\left[\mathbf{E}^{\dagger}(\mathbf{r})\times\mathbf{H}(\mathbf{r})+c.c.\right]d^{3}\mathbf{r}. \end{matrix}$ (16)

In numerical calculation, the electric field and magnetic field are calculated by the finite element method (FEM) eigen solver provided by the software COMSOL Multiphysics. When the electric field and magnetic field of an interface state are calculated and normalized, the group velocity of the interface state can be calculated by numerical integration according to Supplementary Equation (16).

1. Topological routers based on topological rainbow effect

The topological rainbow effect can be used to construct optical routers. As is shown in Supplementary Fig. 2a, line defects are introduced in the deformed region to form a PC waveguide array. When the structure is luminated by a polychromatic light, different frequency components will excite the localized modes at different positions, which couple to different waveguides according to their positions. In order to demonstrate the effect, a two-dimensional PC is used to construct a topological router. The PC consists of air holes with radius *r* = 0.4*a* in a dielectric with index *n* = 5 arranged in a triangular lattice, where *a* is the lattice constant. The PC has a TE bandgap from 0.17 *c*/*a* to 0.30 *c*/*a*. Four line-defect waveguides are introduced for routing, where the distance from the start points of the waveguides to the interface is one lattice constant. In Supplementary Fig. 2b, the calculated results of a 2D FEM simulation for different incident frequencies are presented. For different frequencies, topological photonic states are routed to different output waveguides.

**
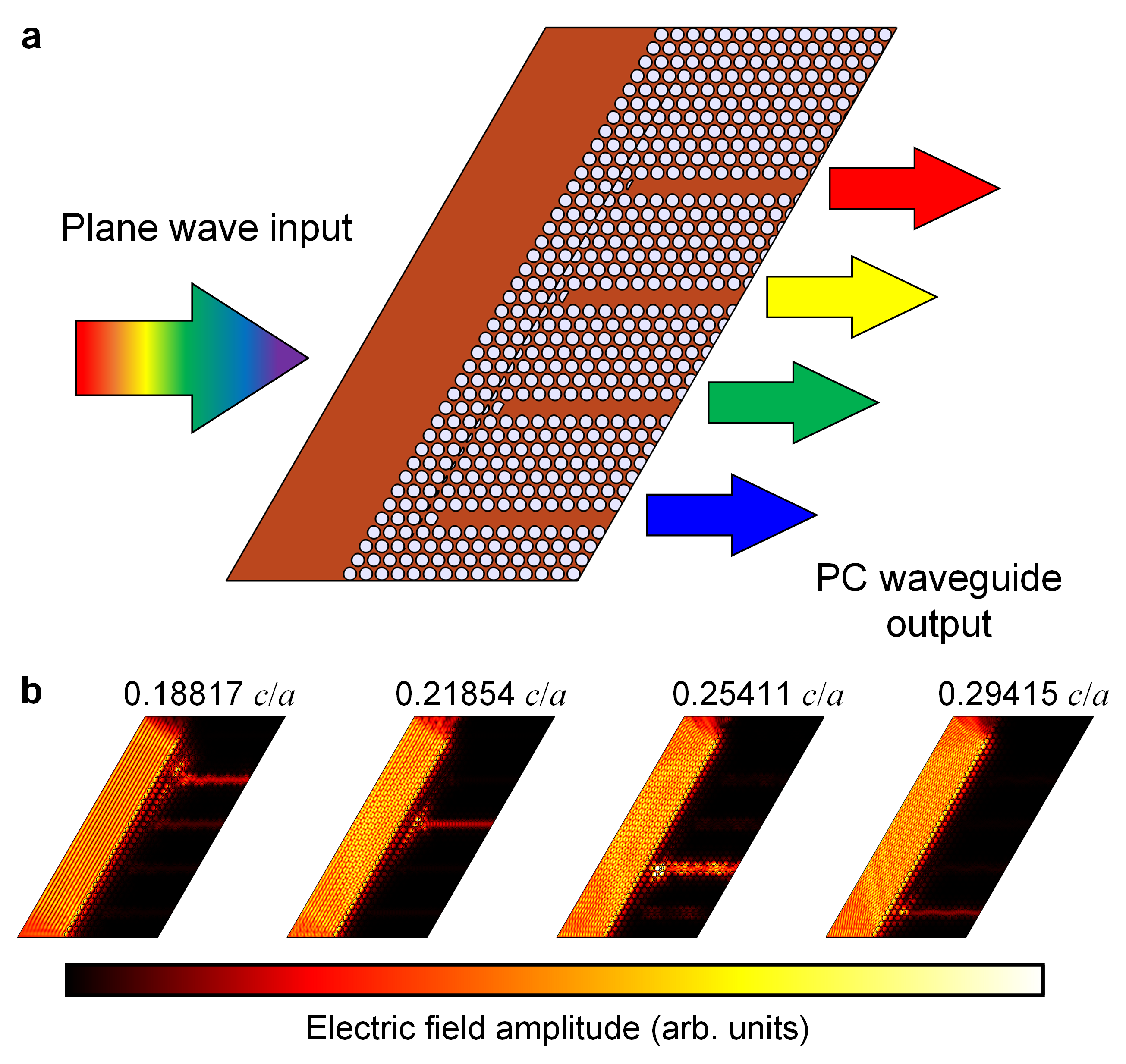
**

**Supplementary Fig. 2. Schematic diagram and calculation results for topological routers.** **a**, The brown domain denotes the dielectric with index *n* = 5, and the white circles denote the air holes with radius *r* = 0.4 *a*, where *a* is the lattice constant. **b**, The distributions of electric field amplitude for different incident frequencies, TE polarization. The frequencies are marked on top of each figure with unit *c*/*a.*

1. Topological rainbow without overlaps of intensity

Limited to the low refractive index of practical materials and laser tunable range in the experiment, the overlaps of the intensity distributions for different wavelengths are not eliminated in the triangular-hole structure and the square-hole structure. However, the mode overlaps can be reduced and even eliminated by designing the bands of PC in principle. As is schematically shown in Supplementary Fig. 3a, if there exist several values of *ξ* satisfying that the frequency ranges of the interface states for different *ξ*s do not intersect with each other, for example, *ξ_i_*, *i* = 1,2,3,4,5 in Supplementary Fig. 3a, one frequency at each frequency intervals between the frequency range of adjacent bands, as the frequencies *ν*_1_,*ν*_2_,*ν*_3_,*ν*_4_ in Supplementary Fig. 3a, so that the intersections of the projected interface bands and the four frequencies dose not overlap with each other. Therefore, the intensity distributions of the four frequencies only overlap with each other in the evanescent mode regions, where the intensity of each mode decays exponentially in space and the overlaps between adjacent modes are small. According to Supplementary Fig. 3a, in order to achieve more small overlap modes in one topological rainbow, the frequency ranges of the interface states should be relatively small compared with the bandgap.

By increasing the refractive index and optimizing the geometry of the unit cell, we theoretically design a topological rainbow structure with large number of small overlap modes. As is shown in Supplementary Fig. 3b, the unit cell of the PC consists of circular holes in a dielectric slab. The lattice of the PC is triangular lattice with lattice constant *a* = 336 nm. The refractive index of the dielectric is *n* = 5.6, the thickness of the slab is *h* = 220 nm, and the radius of the circular hole is *r* = 0.4*a*. The TE-like modes of the bulk bands show a large bandgap from 165.5 THz (1,811 nm) to 252.9 THz (1,185 nm). Next, the intensity distributions of the topological rainbow consisting of the circular hole PC are numerically calculated by FDTD method. Supplementary Fig. 3c shows the top view of the geometric model, where the blue part denotes the dielectric, and the white part denotes the holes. The numbers of the PC layers are *N_a_* = 6, *N_b_* = 9, *N_ξ_* = 20, *N_u_* = 3, and *N_d_* = 6. The light is incident from the waveguide with width *w* = 8 μm. Similar to the structures discussed above, the *x* axis is defined as the central axis of the waveguide, and the *y* axis is defined as the interface of the deformed and undeformed PC.

Supplementary Figure 3d shows that there are at least seven distinguishable wavelengths in the bulk bandgap, which are respectively 1,200, 1,240, 1,277, 1,323, 1,459, 1,606 and 1,772 nm. The corresponding *y* coordinates of the points with maximal intensity are -1,953, -1,619, -1,279, -953, 400, 1,699 and 2,606 nm, respectively. The displacement rate is Δ*y*/Δ*λ* = 7.97, and different modes are separated completely in space due to the highly localized modes. As is shown in Supplementary Fig. 3d, the intensity distributions for the seven wavelengths are separated by the horizontal lines, whose *y* coordinates are -1,750, -1,416, -1,041, -496, 821, and 2,061 nm, respectively. Therefore, by changing the material and optimizing the geometry of the PC, better light separation can be achieved, which shows the flexibility of our method to construct topological rainbow.

**
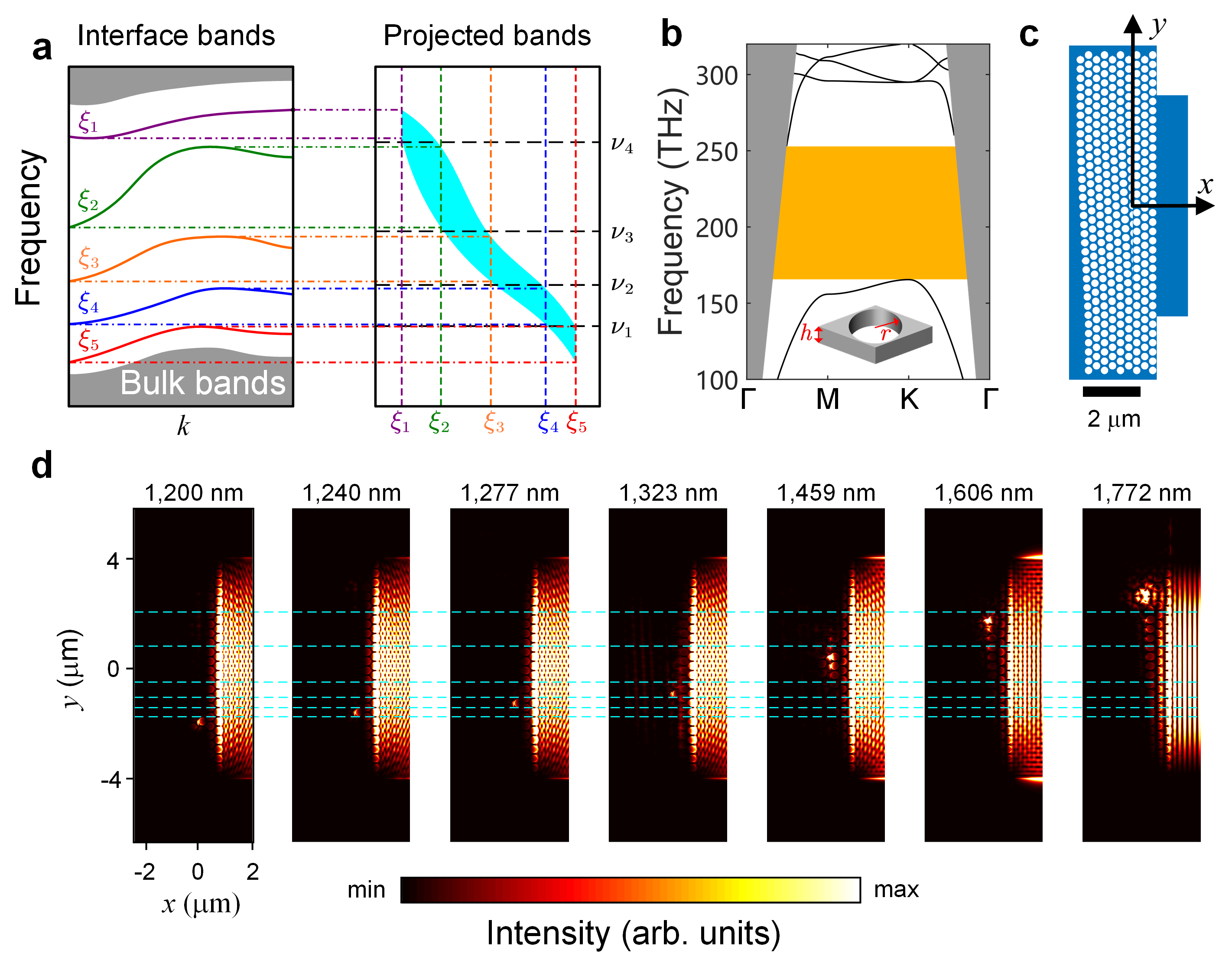
**

**Supplementary Fig. 3. Topological rainbow with small mode overlaps**. **a**, The schematic diagram of the interface bands and projected interface bands of the small overlap modes. **b**, The bulk bands of the TE-like modes. The light cone is marked by the grey region, and the bandgap is marked by the orange stripe. The inset shows the geometry of the unit cell, where the grey part denotes the dielectric with index *n* = 5.6. The parameters are *h* = 220 nm and *r* = 0.4*a*, where *a* is the lattice constant, *a* = 336 nm. **c**, The top view of the geometric structure. The blue part denotes the dielectric, and the white part denotes the air. The light is incident from the waveguide with width *w* = 8 μm. **d**, Intensity distributions for different wavelengths. The cyan lines are parallel to *x* axis and separate different modes.

1. The topological rainbow in higher dimensions

Beside the topological rainbow in 2D system, our method can be generalized to higher dimensions based on a high-dimensional lattice system. Consider a periodic potential in *n* dimensional real space $\tilde{V}(x_{1},x_{2},\ldots,x_{n})=V(\sum_{j=1}^{n} x_{j}\mathbf{a}_{j})$, where **a***_j_* is the *j*-th Bravais lattice vector, $V(\mathbf{r})$ is the periodic potential satisfying $V(\mathbf{r}+\mathbf{a}_{j})=V(\mathbf{r})$, and $\tilde{V}(x_{1},x_{2},\ldots,x_{n})$is the representation of potential in the basis of lattice vectors, which is of period 1 for each argument *x_j_*, *j* = 1, 2, …, *n*. Because of the discrete translational symmetry of the system, the general eigenstates of the periodic system can be expressed in the form of an *n*-dimensional Bloch function $\psi_{m,\mathbf{k}}(x_{1},x_{2},\ldots,x_{n})=\exp(i\mathbf{k}\cdot\mathbf{r})u_{m,\mathbf{k}}(x_{1},x_{2},\ldots,x_{n})$, where **k** is the Bloch wavevector and *m* is the band index. $u_{m,\mathbf{k}}(x_{1},x_{2},\ldots,x_{n})$ is the periodic part with period 1 for each argument *x_j_*, *j* = 1, 2, …, *n*. By defining the reciprocal lattice vectors **b***_j_*, *j* =1, …, *n*, which satisfy $\mathbf{a}_{i}\cdot\mathbf{b}_{j}=2\pi\delta_{ij}$, the Bloch wavevector **k** can be expanded in the basis of reciprocal vectors, which is $\mathbf{k}=\sum_{j=1}^{n} k_{j}\mathbf{b}_{j}$.

Next, similar to the two-dimensional cases, the translational deformation in the direction of **a***_n_* is added to the system and the change of Zak phase along *ξ*-dimension is investigated via the (1+*n*)-dimensional hybrid Wannier functions. For potential $\tilde{V}(x_{1},x_{2},\ldots,x_{n}-\xi)$, the Bloch states are denoted as $\psi_{m,\mathbf{k},\xi}(x_{1},\ldots,x_{n})=\exp(i\chi(\xi))\psi_{m,\mathbf{k}}(x_{1},\ldots,x_{n}-\xi)$, where $\exp(i\chi(\xi))$ is an arbitrary phase factor. The Zak phase along the direction of **b***_n_* and the (1+*n*)-dimensional Wannier function is defined as Supplementary Equation (17) and (18), respectively,

$$\theta_{m}^{\left(\text{Zak}\right)}\left(k_{1},k_{2},\ldots,k_{n-1},\xi\right)=\int_{-1/2}^{1/2} \langle u_{m}\left(k_{1},k_{2},\ldots,k_{n},\xi\right)|i\frac{\partial}{\partial k_{n}}|u_{m}\left(k_{1},\ldots,k_{n},\xi\right)\rangle dk_{n}$$

$$|W_{m}\left(k_{1},\ldots,k_{n-1},R,\xi\right)\rangle=\int_{-1/2}^{1/2} e^{-2\pi ik_{n}R}||\psi_{n}\left(k_{1},\ldots,k_{n},\xi\right)\rangle dk_{n}$$

where $\psi_{m,\mathbf{k},\xi}(x_{1},\ldots,x_{n})$ and $u_{m,\mathbf{k},\xi}(x_{1},\ldots,x_{n})$are denoted by Dirac brackets $|\psi_{m}\left(k_{1},\ldots,k_{n},\xi\right)\rangle$ and $|u_{m}\left(k_{1},\ldots,k_{n},\xi\right)\rangle$, respectively. By direct computation, the Zak phase is related to the expectation value of *x_n_* of the hybrid Wannier functions, as is shown in Supplementary Equation (19).

$$\begin{matrix} \theta_{m}^{\left(\text{Zak}\right)}\left(k_{1},k_{2},\ldots,k_{n-1},\xi\right) \\ = 2\pi\langle W_{m}\left(k_{1},\ldots,k_{n-1},R,\xi\right)|\hat{x}_{n}|W_{m}\left(k_{1},\ldots,k_{n-1},R,\xi\right)\rangle\mathrm{mod} 2\pi\end{matrix}$$

Therefore, when *ξ* changes for one period, the Zak phase along bn dimension will change for 2*π*, resulting in a non-zero Chern number 1 of the subspace (*k_n_*, *ξ*).

For interface states, the deformed-undeformed interface is constructed by truncating the deformed crystal and the undeformed crystal along the crystal plane perpendicular to **b***_n_*, and joining the two parts together. The joint structure preserves (*n* – 1)-dimensional translational symmetries along the lattice vectors **a**_1_, **a**_2_, …, **a***_n_*_-1_, and the reciprocal lattice vectors are projections of **b**_1_, **b**_2_, …, **b***_n_*_-1_ onto the interface. According to bulk-edge correspondence, there exists interface states when *ξ* traverses the whole period, whose dispersion line for fixed $(k_{1},k_{2},\ldots,k_{n-1})$ crosses the whole bulk bandgap. Supplementary Figure 4 schematically illustrates the idea in 2D and 3D lattices. For 2D lattices, when the undeformed and deformed lattices are joined together (as is shown in Supplementary Fig. 4a), the interface supports 1D interface states whose dispersion relations vary with *ξ*, as is schematically shown in Supplementary Fig. 4b. Similarly, for 3D lattices, an 2D interface is formed when joining the undeformed lattices and undeformed lattices, as is marked by the dashed frame in Supplementary Fig. 4c, and 2D interface states can be observed. The topology in (*k_n_*, *ξ*) space ensures the existence of topological interface states in synthetic space, that is, the dispersion line of fixed transverse wavevector (*k*_1_, *k*_2_) exists and crosses the bandgap when *ξ* changes by a period, as is shown in the left part of Supplementary Fig. 4d. For another thing, when the dispersion bands are viewed in the wavevector space (the right part of Supplementary Fig. 4d), the dispersion of interface states move downwards from the upper bulk band to the lower bulk band when *ξ* changes by a period.


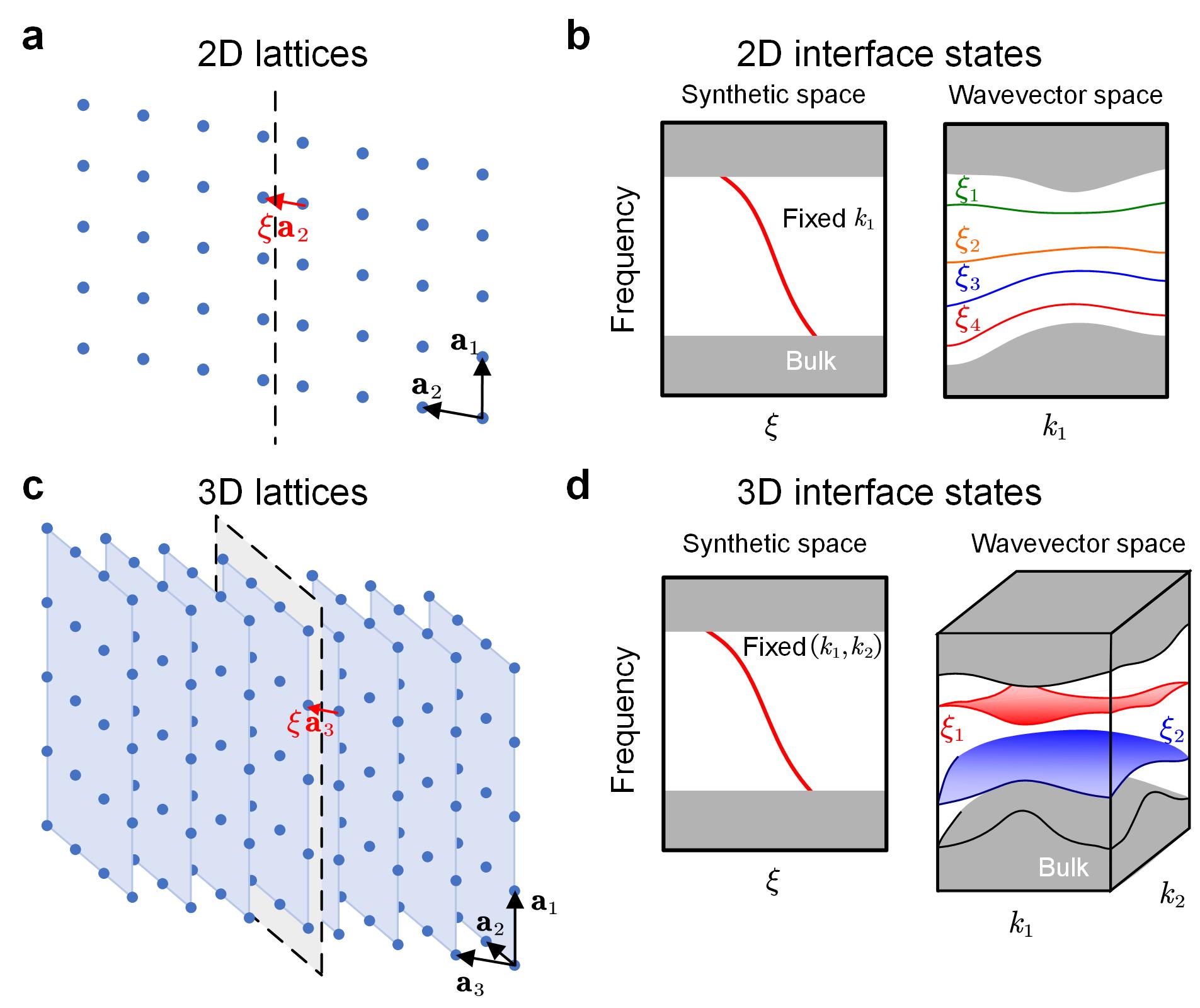


**Supplementary Fig. 4. Topological rainbow in 2D and 3D lattices**. **a**, The schematic diagram of the deformed-undeformed interface in 2D lattices, where the dashed line denotes the interface. **b**, The schematic graph of the bands of 2D interface states, where the colored bands denote the interface bands and the grey parts denote the bulk bands. **c,** The schematic diagram of the deformed-undeformed interface in 3D lattices, where the dashed frame denotes the interface. **d,** The schematic graph of the bands of 3D interface states, where the colored bands denote the interface bands and the grey parts denote the bulk bands.

In order to demonstrate the topological rainbow effect in a 3D lattice, a gapped 3D topological rainbow is constructed based on a diamond lattice PC ^4^. The structure is constructed by etching spherical holes in a dielectric on each site of a diamond lattice (Supplementary Fig. 5a). Supplementary Figure 5b shows the geometry of four sites. For permittivity *ε* = 13 and the radius of the sphere *r* = 0.325*a*, the dispersion bands show a bulk bandgap from 0.492 *c*/*a* to 0.663 *c*/*a*, as is shown in Supplementary Fig. 5c. Under the bulk bandgap, there are two bulk bands. Therefore, by modulating parameter *ξ*, two interface bands are expected. Next, by constructing the undeformed-deformed interface along the plane spanned by **a**_1_, **a**_2_, a 2D interface with 2D triangular lattice is constructed. The bands of the spliced structure are shown in Supplementary Fig. 5d, where two interface bands move from the second bulk band to the first bulk band as *ξ* increases by a period, consistent with the prediction of topology.


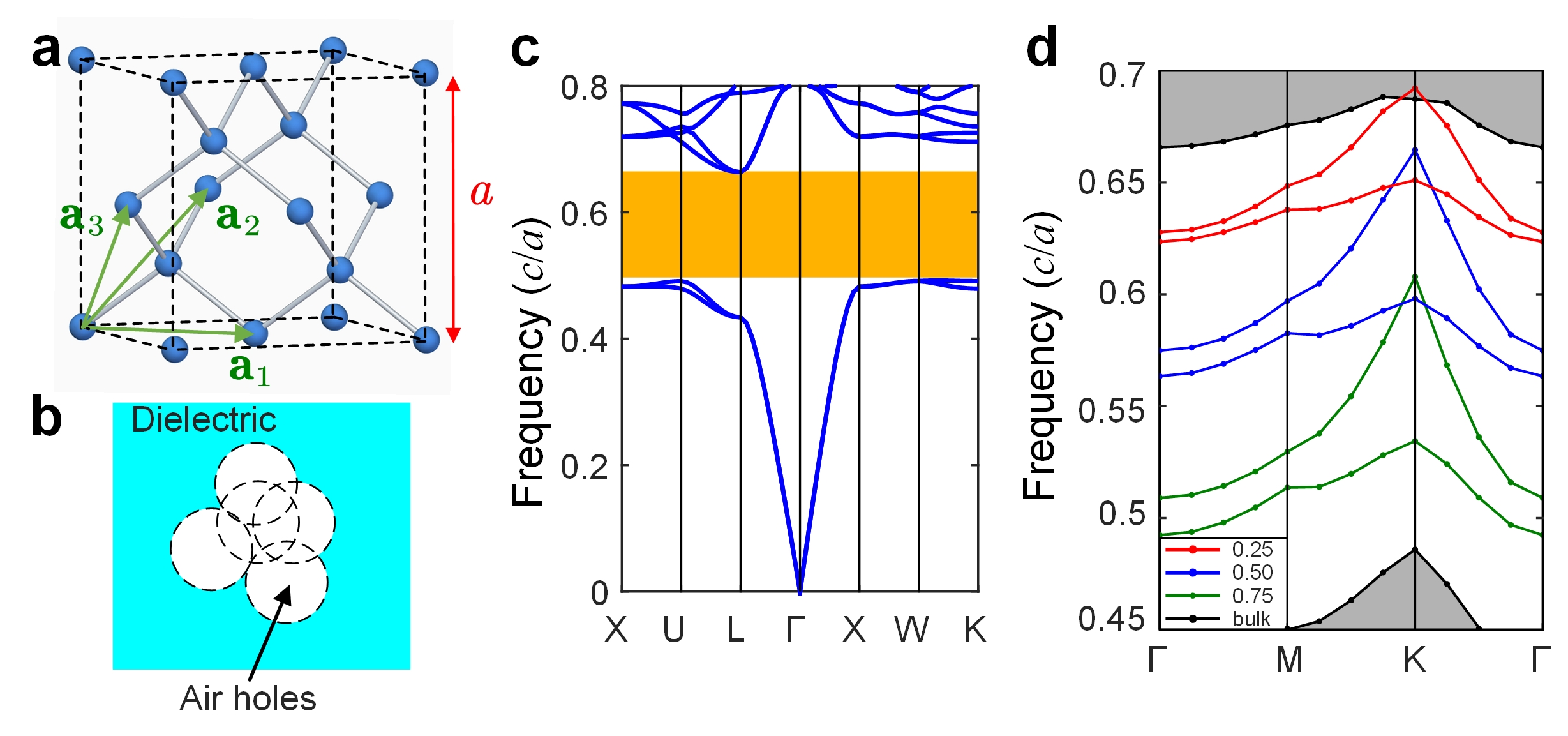


**Supplementary Fig. 5. Geometric structure and bands of the diamond lattice PC**. **a**, The crystalline structure of a diamond lattice. The three Bravais vectors **a**_1_, **a**_2_, **a**_3_ and the lattice constant *a* are marked. **b**, The geometry of 5 adjacent sites. The cyan part denotes the dielectric, and the white part denotes the spherical air holes. **c**, Bulk bands, where the orange stripe marks the bandgap. **d**, Interface bands, where the grey parts denote the bulk bands, and the colored lines denote the interface bands for different *ξ*.

Next, by gradually modulate the amount of *ξ* along **a**_1_ direction, as is shown in Supplementary Fig. 6a, frequency-dependent conductive channels are constructed. Because in modulated structure, *ξ* is dependent of *x*_1_, but independent of *x*_2_, the interface states with different frequencies will split in the direction of **a**_1_, but are allowed to propagate in the direction **a**_2_, forming conductive channels whose positions depend on frequencies. We numerically calculated the intensity distributions when a plane wave source is incident along the interface. The wavevector of the incident wave lies in the interface (dashed frame in Supplementary Fig. 6a) and perpendicular to **a**_1_. We define the polarizations of incident light with electric vectors perpendicular or parallel to the interface plane as perpendicular polarization or parallel polarization respectively, and calculate the intensity distributions on the interface plane. For numerical calculation, a 3D rainbow with lattice numbers *N*_1_ = 21, *N*_2_ = 6, *N*_3_ = 3 defined in Supplementary Figure 6a is constructed, the geometric diagram of which is shown in Supplementary Figure 6b. Supplementary Figure 6c, 6d show the intensity distributions for perpendicular polarized light and parallel polarized light respectively. The *x* and *y* axes are perpendicular and along lattice vector **a**_1_, respectively. The frequencies of incident wave are marked on top of each figure, and the unit is *c*/*a*. For both perpendicular polarized states and parallel polarized states, the conductive channels (marked by the green dashed lines) move to the position with smaller *ξ* when the frequency increases, which is consistent with the analysis of topology. Comparing the results of perpendicular and parallel polarized sources, however, the results are slightly different. For perpendicular polarized source, there are clear separated channels that supports interface states, while for parallel polarized sources, the channels are not as evident as the states under perpendicular polarized source. This is resulted from the mode mismatch between the propagating modes in the channels and the incident plane waves. When parallel polarized light is incident into the structure, most of the energy couples into evanescent modes, and only a little energy excites the interface modes.


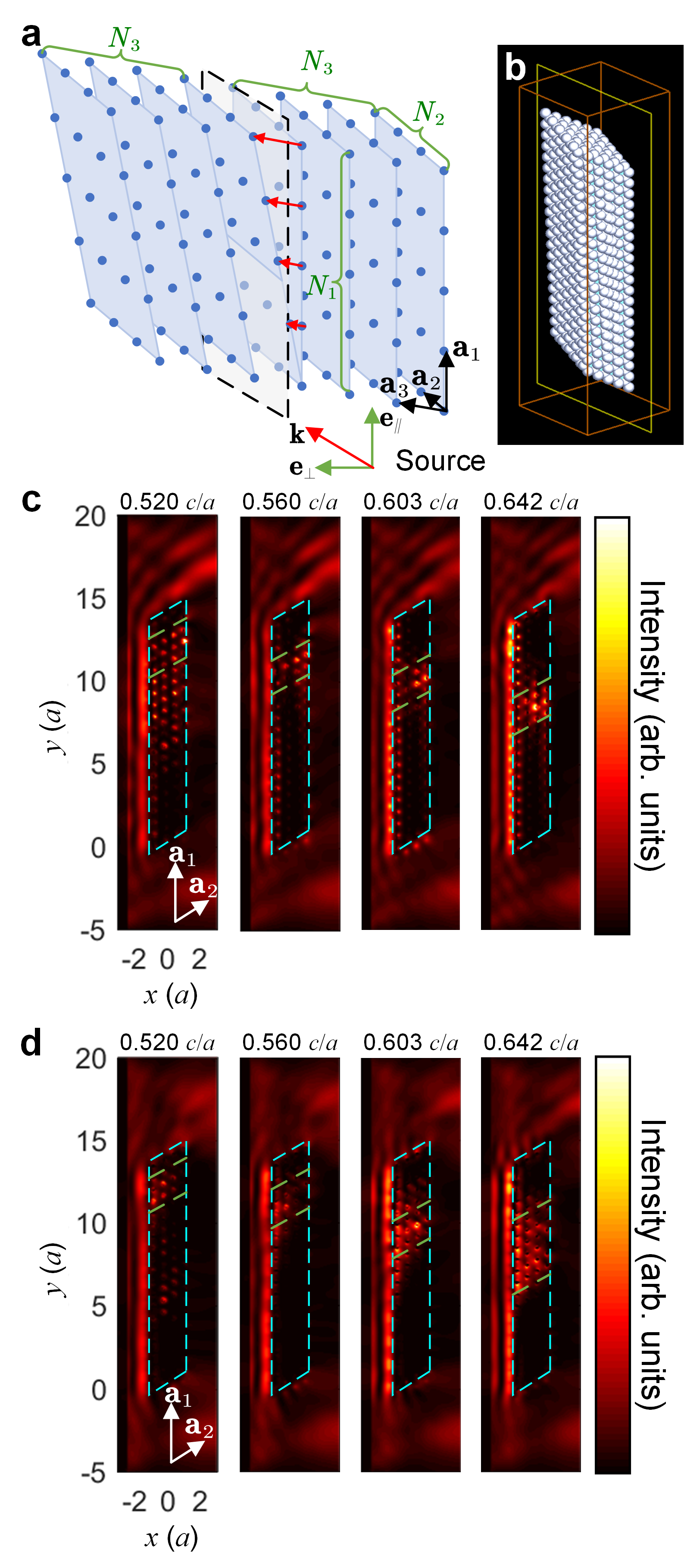


**Supplementary Fig. 6. Calculated results of a 3D topological rainbow**. **a**, The schematic diagram of the 3D topological rainbow. **b**, The geometric structure of the 3D topological rainbow, where the grey spheres are air holes, and the cyan material denotes the dielectric. The deformed-undeformed interface is marked by the yellow frame. The shape parameters are *N*_1_ = 21, *N*_2_ = 6, *N*_3_ = 3 **c**, **d**, The calculated results of interface intensity distributions for perpendicular (**c**) and parallel (**d**) polarized light. The dashed cyan lines mark the profile of the structure. The length unit is lattice constant *a*, and the frequency is marked on the top of each figure, the unit is *c*/*a*. The green dashed lines mark the conductive channels.

1. Sample fabrication methods and SEM images in a large scale

The samples are fabricated on a common silicon-on-insulator (SOI) chip that includes a 220 nm-thick silicon layer and a 2 μm-thick SiO_2_ layer. The ultraviolet lithography (model: MA6, SN201208226) and inductively coupled plasma etching system (OXFORD PlasmaPro 100 Cobra 180) were adopted to fabricate the input waveguide with a width of 8 μm and a length of 1 mm, and then the facet of waveguides was polished by the custom face grinding and polishing machine (model: FM-420). The SEM image for the waveguides is shown in Supplementary Fig. 7a.

The patterns of the nano-structures were fabricated by using the focused-ion-beam (FIB) system (Helios G4 UC of FEI company) with an ion beam current of 40 pA. The SEM images of the sample with triangular air holes and square air holes in a large scale are shown in Supplementary Fig. 7b and 7c, respectively.


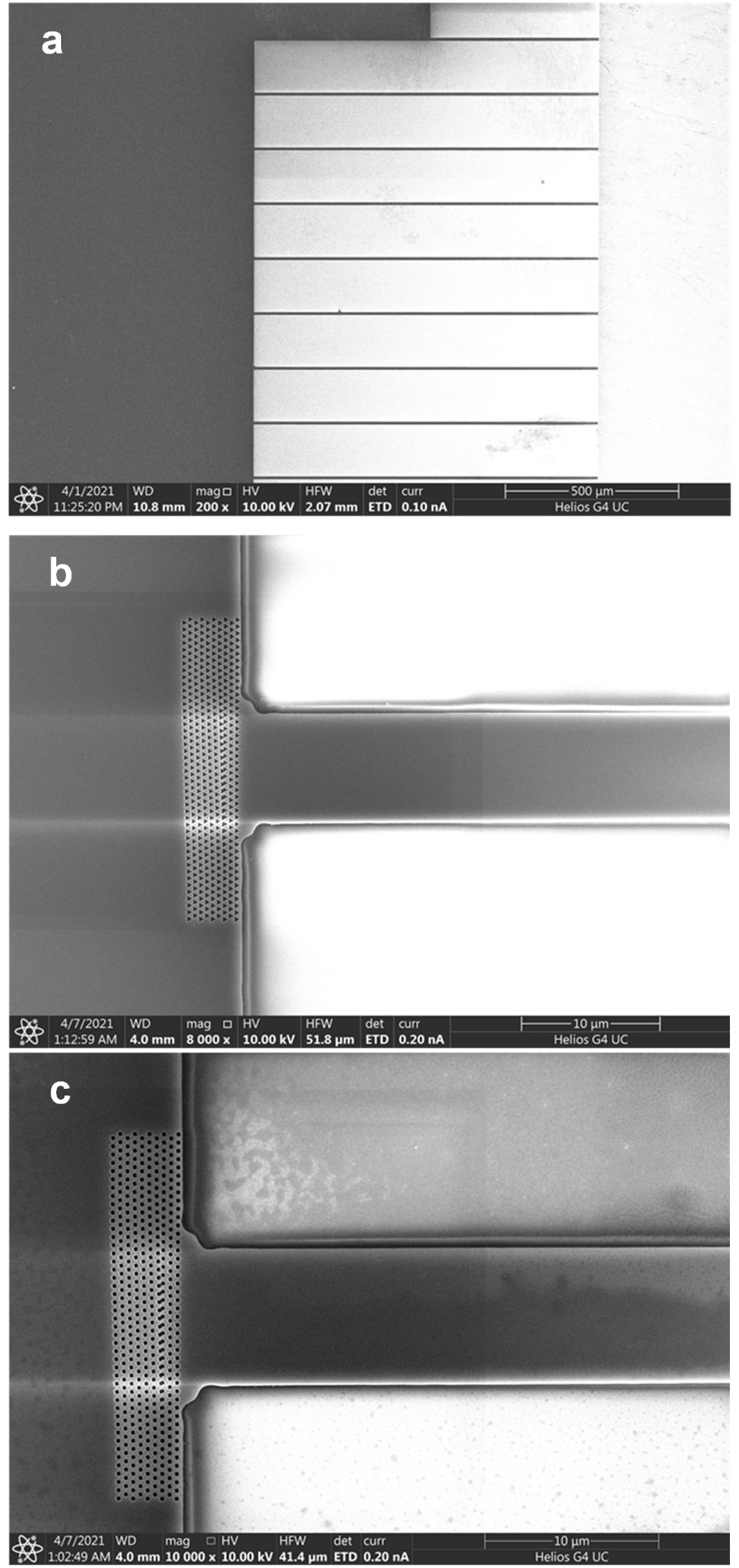


Supplementary Fig. 7. SEM images of samples in a large scale. a, The waveguide arrays have a length of 1 mm and a width of 8 μm. b, SEM image of the triangular-hole sample in a large scale; c, SEM image of the square-hole sample in a large scale.

1. Different variation rate of the synthetic parameter *ξ*

Apart from designing the bulk photonic bands, the variation rate of the translational parameter *ξ* along the interface is another factor that will influence the light separating ability of the topological rainbow. The idea of constructing a rainbow device using the translational synthetic dimension is to modify the translational parameter along the interface. When the variation rate is small enough, the deformed PC can be viewed as a uniformly translationally deformed PC with a certain local synthetic parameter. However, as for a rainbow device, the variation rate cannot be infinitely small, which will lead to an infinite size of device. Therefore, it is important to choose a moderate rate of variation.

In the main text, the geometric parameter *N_ξ_* is defined to characterize the variation rate of *ξ*, as is shown in Fig. 1**a** in the main text. The parameter *N_ξ_* equals the number of cells along the interface while *ξ* changes for a period. In order to show the influence of the different rates of variation, the calculated intensity distributions of the structures with different *N_ξ_*’s are shown in Supplementary Fig. 8, where the results of the structures with *N_ξ_* = 10 (**a**, **b**), *N_ξ_* = 30 (**c**, **d**) and *N_ξ_* = 50 (**e**, **f**) are shown respectively. The structural parameters are the same as the main text except *N_ξ_*. The width of the incident waveguide is kept as 8μm, and the size of the source is hard to enlarge, because a wider waveguide suffers more interference of the high-order modes, which will shrink the width of the luminance. For a larger rate of variation (*N_ξ_* = 10), although the size of the mode profile of each interface mode is small, the spatial movements of interface states against the variation of the frequency is also small. When wavelength changes from 1,540 nm to 1,630 nm, the total amount of the movement of interface modes is less than 1 μm, which is hard to detect in experiment. For a smaller rate of variation (*N_ξ_* = 10), the interface modes stretch correspondingly, and the size of the mode profile is comparable to the width of the incident waveguide, making it hard to excite the modes that are away from the source. Considering both the spatial dispersion rate of the interface modes and the width of the source, the parameter *N_ξ_* = 30 (main text and Supplementary Figs. 8**c**, 8**d**) is chosen.


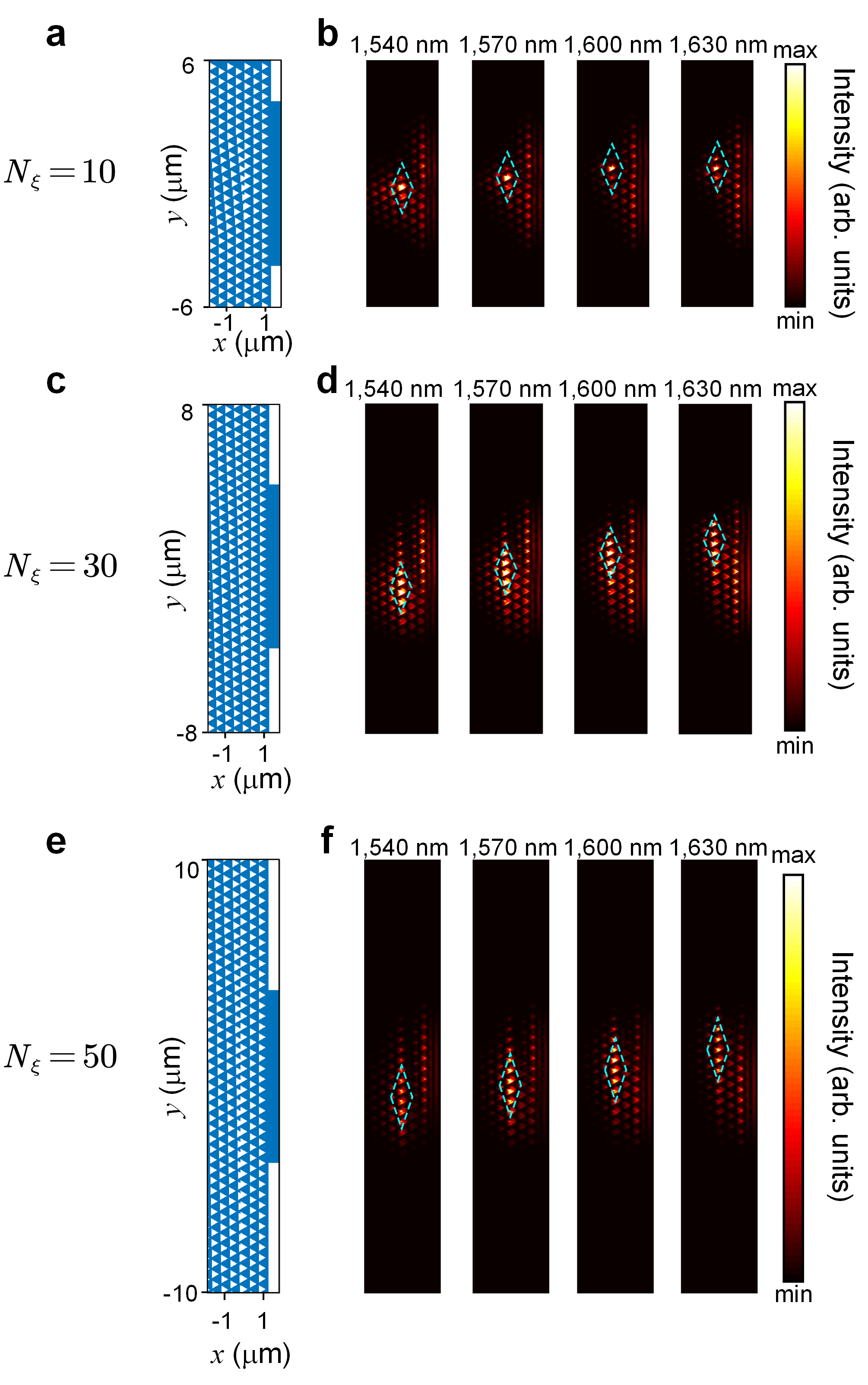


Supplementary Fig. 8. The geometry and calculated intensity distributions for structures with different variation rate of displacement vectors. a, b, Geometry (a) and intensity distributions (b) of the structure with *N_ξ_* = 10. c, d, Geometry (c) and intensity distributions (d) of the structure with *N_ξ_* = 30. e, f, Geometry (e) and intensity distributions (f) of the structure with *N_ξ_* = 50. The position with maximal intensity is marked by the cyan dashed rhombuses. The incident wavelengths are marked on the top of each figure in the unit μm.

1. Topological rainbow for square holes structures

The dispersed interface states in the topological rainbow result from the non-trivial topology of translational deformation dimension, and the existence of these interface states is not influenced by the dispersion bands in geometric dimension. Generally, any gapped photonic crystals will show similar interface states. In this section, a topological rainbow with different geometric structure will be discussed. As is shown in Supplementary Fig. 9a, the unit cell consists of a square hole in a triangular lattice. The diagonals of the square hole coincide with the diagonals of the rhombus unit cell. The geometric parameters are marked in the inset of Supplementary Fig. 9a. For this model, the side length is *l* = 0.467*a*, where *a* = 475 nm is the lattice constant, and the thickness of the silicon wafer is 220 nm. Supplementary Fig. 9a shows the bulk bands of TM modes. The band structure shows a bandgap ranging from 168.2 THz (1,782 nm) to 205.0 THz (1,462 nm). Next, we join the translational deformed structure and the undeformed structure, as is shown in the inset of Supplementary Fig. 9b, and calculate the dispersion bands of the supercell. The dispersion bands are shown in Supplementary Fig. 9b. The green and grey regions denote the projective bulk bands and the light cone respectively, and the colored lines denote the dispersion bands of the interface states. Although the geometric structure and bulk bands of the square-hole structure are quite different from the triangular-hole structure in the main text, the dispersion of interface bands show the common behavior. When *ξ* increases from 0 to 1, the dispersion bands move downwards from the second bulk band to the first bulk band because of the topology in *ξ* dimension.


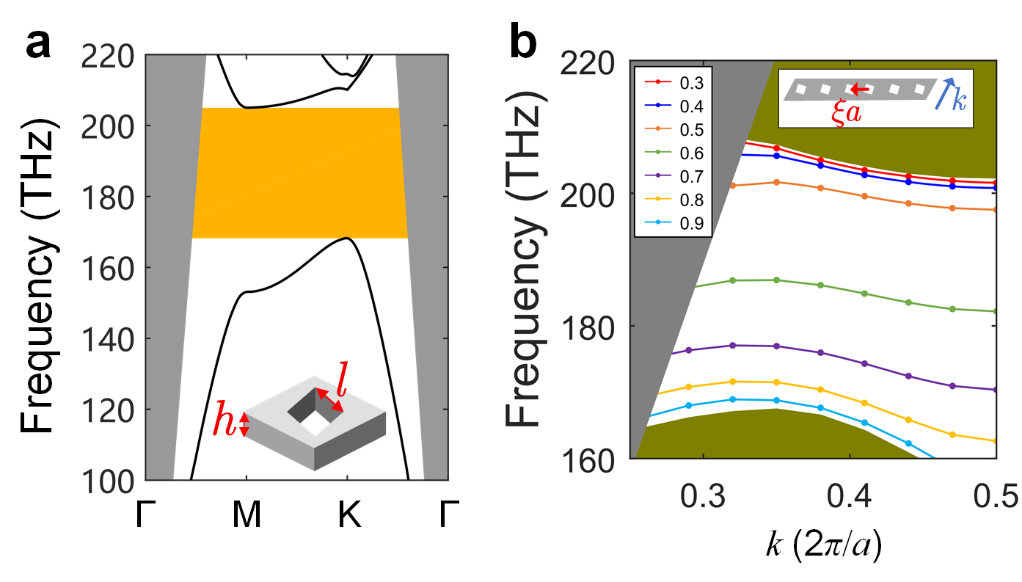


Supplementary Fig. 9. Bulk and interface bands for square-hole structure. a, Bulk dispersion bands of the TE modes. The inset shows the geometric structure. The bandgap and light cone are marked with orange and grey regions respectively. b, Interface dispersion bands of the TE modes. The super lattice is shown in the inset, with the displacement and the direction of Bloch wave vector marked. The green and grey regions denote the projective bulk bands and the light cone respectively, and the lines with different colors denote the interface bands with different *ξ*.

Similar to the triangular-hole structure, we numerically calculated the electric field distributions when the structure is incident by an 8 μm-wide waveguide with different frequencies, and experimentally fabricated and tested the structure with the same equipment as the triangular-hole structure. Supplementary Fig. 10a shows the SEM image of the whole sample and the zoom-in view of the interface marked by red dashed rectangle. The barrier and dispersing regions are colored by red and green respectively. The experimental results are shown in Supplementary Figs. 10b and 10c. Supplementary Fig. 10b shows the topography of the sample, detected by the s-SNOM system, and Supplementary Fig. 10c shows the light intensity distributions for the four wavelengths. The positions with maximal intensity are also marked. When the wavelength increases, the interface modes also move towards *y* direction, with *y* coordinates -756, -676, 253, 1,182 nm, and the displacement rate is 32.3. The experimental results are also compared with the calculated results. Supplementary Fig. 10d shows the top view of the FDTD model, where the blue region denotes the silicon wafer, and the white regions denotes the holes. Supplementary Fig. 10e shows the FDTD results of the light intensity distributions. The positions with maximal intensity are marked with cyan dashed rhombuses. When the wavelength increases, the position of the maximal intensity moves toward *y* direction. For wavelengths 1,530, 1,550, 1,570, 1,590 nm, the *y* coordinates are -1,566, -636, -156, 774 nm, and the displacement rate is 39.0. The movement of interface states when wavelength changes is consistent with the dispersion bands of topological interface states, where the range of *ξ* increase when frequency decrease, same as the triangular-hole structure shown in the main text.

In conclusion, the topology of translational deformation is not influenced by the unit cell structures. For different gapped PC, the topological rainbow can be constructed by graded translational deformation. Because the topology of lattice translational deformation is independent of the specific geometry structure, the geometric structure can be finely tuned to design the dispersion bands for a specific application, while the topological interface states still exist.


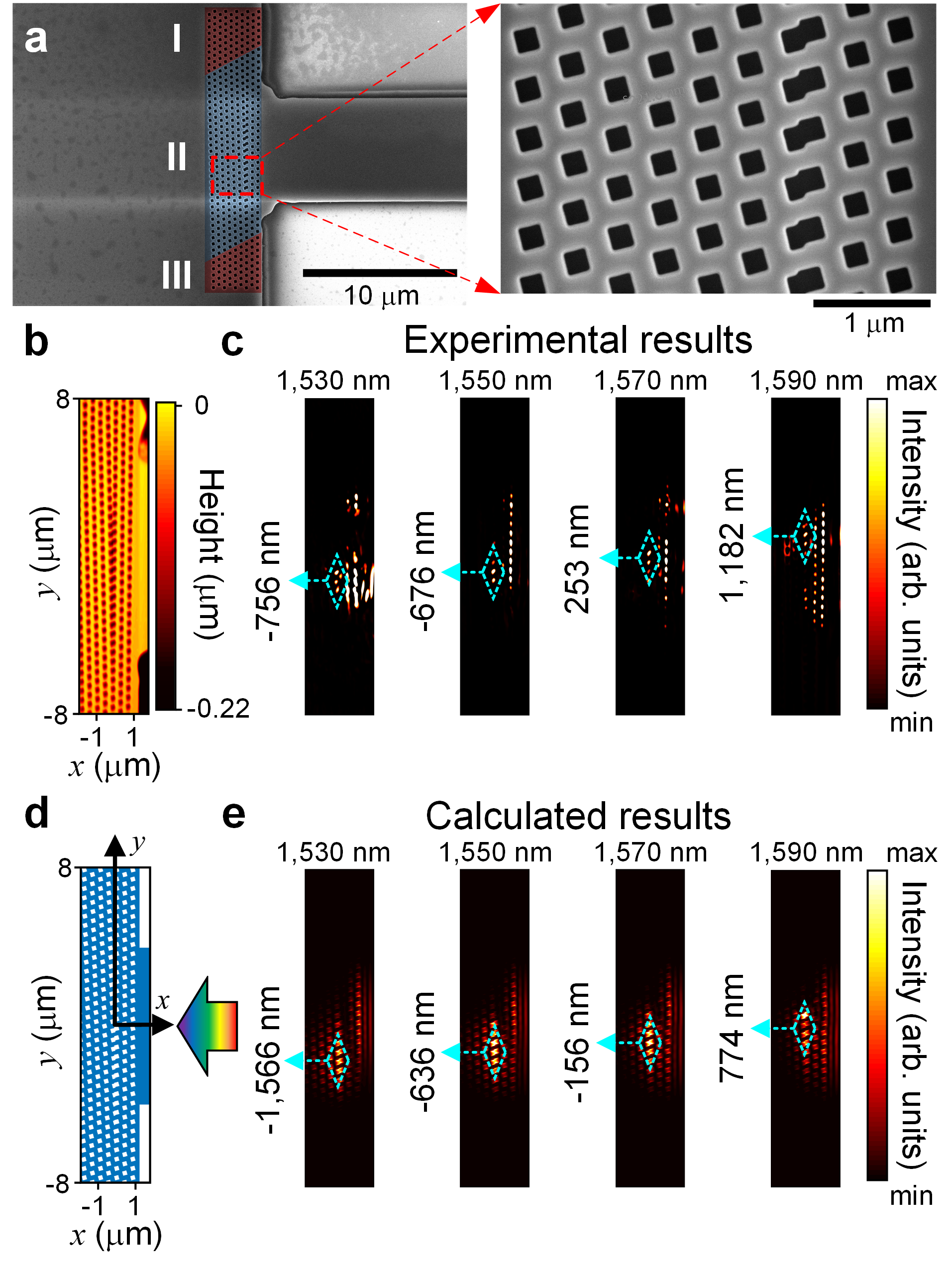


Supplementary Fig. 10. Numerical and experimental results of square-hole structure. a, SEM images. b, The topographic image of the sample. c, The experimental results of the intensity distributions at the surface of the sample. d, The top view of the FDTD model. e, The FDTD results of intensity distributions for different wavelengths. The positions with maximal intensity are marked by the cyan dashed rhombuses, and the corresponding *y* coordinates are marked on the left. In c and e the wavelengths of incident light are marked on the top of the electric field distributions, and the unit is nm.

References and notes

1. Zak, J. Berry’s phase for energy bands in solids. *Phys. Rev. Lett.* **62**, 2747–2750 (1989).

2. Politzer, P. & Murray, J. S. The Hellmann-Feynman theorem: a perspective. *J. Mol. Model.* **24**, 266 (2018).

3. Raghu, S. & Haldane, F. D. M. Analogs of quantum-Hall-effect edge states in photonic crystals. *Phys. Rev. A* **78**, 033834 (2008).

4. Joannopoulos, J. D., Johnson, S. G., Winn, J. N. & Meade, R. D. *Photonic Crystals: Molding the Flow of Light*. (Princeton University Press, Princeton, 2008).
